# Exploiting Cation Structure and Water Content in Modulating the Acidity of Ammonium Hydrogen Sulfate Protic Ionic Liquids

**DOI:** 10.1021/acs.jpclett.3c03583

**Published:** 2024-02-22

**Authors:** Anton E. J. Firth, Pedro Y. S. Nakasu, Jason P. Hallett, Richard P. Matthews

**Affiliations:** † Department of Chemical Engineering, Imperial College London, London SW7 2AZ, U.K.; ‡ Department of Bioscience, School of Health, Sports and Bioscience, University of East London, Stratford, London E15 4LZ, U.K.

## Abstract

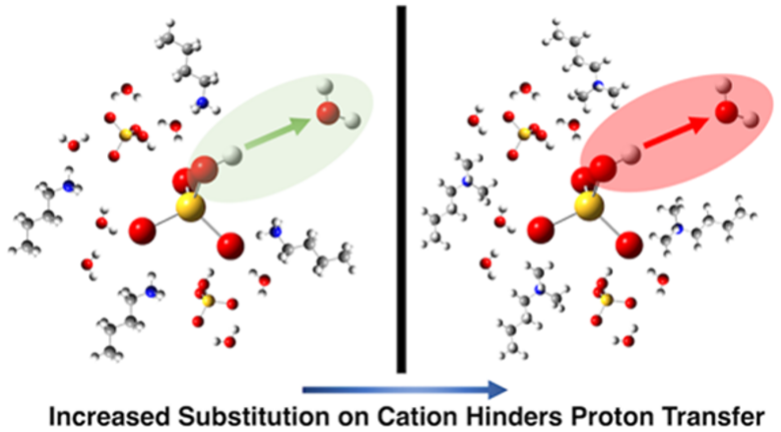

In this paper, we investigated the effect of cation structure and water content on proton dissociation in alkylammonium [HSO_4_]^−^ protic ionic liquids (ILs) doped with 20 wt % water and correlated this with experimental Hammett acidities. For pure systems, increased cation substitution resulted in a reduction in the number of direct anion–anion neighbors leading to larger numbers of small aggregates, which is further enhanced with addition of water. We also observed spontaneous proton dissociation from [HSO_4_]^−^ to water only for primary amine-based protic ILs, preceded by the formation of an anion trimer motif. Investigation using DFT calculations revealed spontaneous proton dissociation from [HSO_4_]^−^ to water can occur for each of the protic ILs investigated; however, this is dependent on the size of the anion aggregates. These findings are important in the fields of catalysis and lignocellulosic biomass, where solvent acidity is a crucial parameter in biomass fractionation and lignin chemistry.

Controlling proton activity in solvent systems is of fundamental importance in chemical and biochemical catalytic processes,^1−5^ in protein structure and function regulation,^6−9^ in biomass degradation,^10−12^ and in electrochemical energy conversion.^13−15^ In aqueous environments, proton dissociation requires active involvement of water molecules via formation of hydronium (H_3_O^+^) or hydroxide (OH^–^) ions which are enabled by structural reorganization and hydrogen-bonding patterns, facilitating subsequent intermolecular proton transfer through the solvent.^16−20^ Likewise, restructuring of the solvent environment and consequent formation of ion chemical gradients facilitate the dissociation and exchange of protons in anhydrous environments such as phosphoric acid,^16^ liquid imidazole,^21^ anhydrous ionic liquids (ILs),^22−24^ and mixtures composed of ILs and molecular species.^25−27^ However, acid dissociation and proton transfer in protic ILs doped with water have been less well investigated at the molecular level.

Protic ILs are organic salts that possess dissociable protons on the cation and/or the anion. This composition facilitates unique properties in anhydrous protic ILs as the ionic components act both as hydrogen bond donors and acceptors and as potential Brønsted acids.^28,29^ This positions protic ILs as ideal alternatives to organic solvents in water/organic mixtures for acid-catalyzed reactions. Recently, enhanced proton exchange in anhydrous protic ionic liquids was revealed to reduce potential loss at the electrode.^22^ Moreover, variation in the concentration of water in [HSO_4_]^−^ based protic ILs has been shown to impact solution acidity and expedite lignin depolymerization via hydrolysis and dehydration reactions.^10,11^ Furthermore, the acidities of neutral acids–diluted in ILs–have been found to be significantly affected by the structure of the aprotic/protic IL cation, with a direct correlation identified between increased acidity and the number of acidic protons on the cation.^30^ Consequently, we undertook this investigation to explain the role of cation structure and water content on the proton dissociation and associated acidity of aqueous alkylammonium [HSO_4_]^−^ based protic ILs.

We consider four alkylammonium [HSO_4_]^−^ protic ILs, each doped with 20 wt % water (Figure 1A). The systems investigated account for varying degrees of N atom substitution, from primary to tertiary, on the cation and density effects. This provides a template for better understanding acidities as a function of the number of N–H hydrogen bond donors on the cation and additional hydrogen bonding interaction sites made available by water molecules which can further enhance or moderate proton transfer and accordingly solution acidity. We employed a multiscale computational approach, including classical molecular dynamics (MD), *ab initio* molecular dynamics (AIMD), and density functional theory (DFT), to provide an atomistic rationalization of the effect of cation structure and water content and correlated the simulation findings with the experimentally measured Hammett acidities.

**Figure 1:**
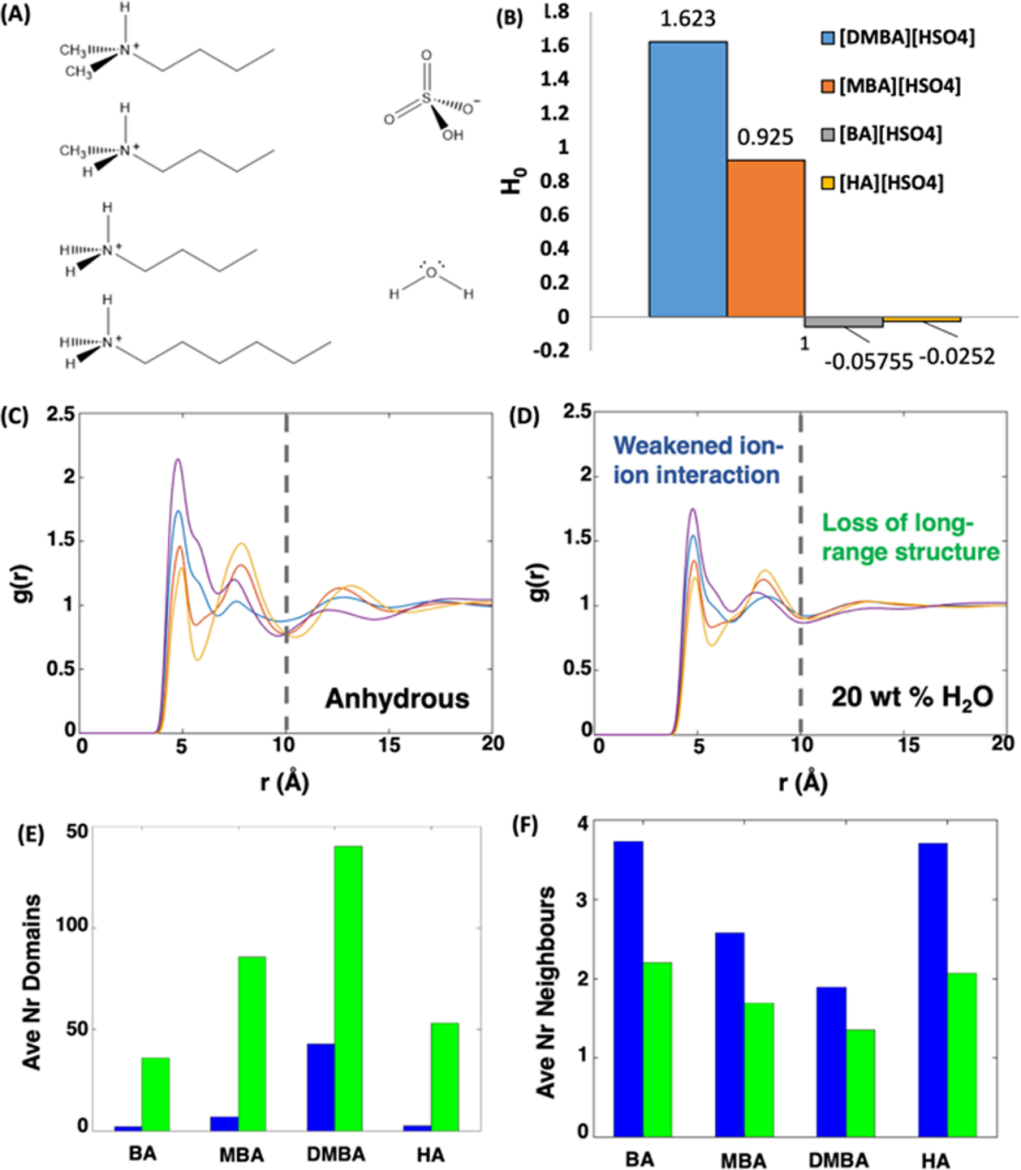
(A) Ionic and molecular components. The cations are from top to bottom *N*,*N*-dimethyl-*N*-*n*-butylammonium (DMBA), *N*-methyl-*N*-*n*-butylammonium (MBA), butylammonium (BA), and hexylammonium (H). (B) Hammett acidities of alkylammonium [HSO_4_]^−^ protic ILs with an acid-to-base ratio of 1.00. All systems contain 20 wt % water. (C) Anion(S)–anion(S) pair radial distribution functions for anhydrous and (D) 20 wt % water containing protic ILs; [BA][HSO_4_] shown in blue, [HA][HSO_4_] in purple, [MBA][HSO_4_] in orange, and [DMBA][HSO_4_] in yellow. (E) Average number of anion–anion domains and (F) average number of immediate [HSO_4_]^−^ neighbors. Plots are colored for anhydrous (blue) and (green) protic ILs in the presence of 20 wt % water.

The array of ionic and hydrogen bonding interactions that persist in ILs ensures that these solvent systems are chemically and structurally complex.^31−37^ This makes the determination of acidity in ILs via traditional aqueous or nonaqueous acid–base theory challenging. Consequently, both protic and aprotic IL acidities are generally expressed using the Hammett acidity function (*H*_0_)^38^ which facilitates direct measurement of acidity while avoiding solvent leveling effects (i.e., limiting of the effect of excess strong acid and base species by the presence of the solvent) and allowing for comparison between solvents. *H*_0_ values for each of the investigated alkylammonium [HSO_4_]^−^ protic ILs doped with 20 wt % water with an acid–base ratio of 1.00 are presented in Figure 1B. The graph corresponds to an acidity order of [HA][HSO_4_] ≈ [BA][HSO_4_] > [MBA][HSO_4_] > [DMBA][HSO_4_], with [HA][HSO_4_] and [BA][HSO_4_] revealed as the strongest acids and [DMBA][HSO_4_] the weakest acid. This indicates that the proton donating propensity and acidities of investigated alkylammonium [HSO_4_]^−^ protic ILs doped with 20 wt % water correlate directly with the degree of substitution on the cation, i.e., protic ILs based on primary amines afford the highest acidities. This result cannot be predicted using aqueous acidity theory, considering that the anion is expected to be the dominant acidic species in [HSO_4_]^−^ based protic ILs with a p*K*_a_ of 2, and the p*K*_a_s of the constituent amines’ conjugate acids in water are similar (between 10.02 and 10.56^39,40^) and do not follow the same acidity trend. Additionally, we note that each of the investigated alkylammonium [HSO_4_]^−^ protic ILs is significantly more acidic than their imidazolium-based analogues. *H*_0_ values of 1.99 and 2.08 have been previously reported for anhydrous 1-methylimidazolium hydrogen sulfate ([Hmim][HSO_4_]) and 1-butyl-3-methylimidazolium hydrogen sulfate ([bmim][HSO_4_]),^41^ compared to *H*_0_ values of 1.62 and −0.03 we obtain for our [DMBA][HSO_4_] and [HA][HSO_4_] samples doped with 20 wt % water. Correspondingly, although the anion facilitates acid dissociation, the varying strengths of observed acidities appear to be highly affected by the nature of the cation and the presence of water.

To elucidate the origin of increased acidity found in our alkylammonium [HSO_4_]^−^ protic ILs doped with 20 wt % water, we conducted a series of *H*_0_ measurements for select systems employing varying acid–base ratios. On addition of excess base (amine) to [BA][HSO_4_], we find that the excess base is fully protonated by [HSO_4_]^−^ anions, resulting in varying [HSO_4_]^−^/[SO_4_]^2–^ anion mixtures and consequently reduced solution acidities (Figure S1). This supports our hypothesis that the increased acidity of the primary alkylammonium [HSO_4_]^−^ protic ILs systems originates from the [HSO_4_]^−^ anion and does not originate from the proton on the alkylammonium cation. Moreover, the addition of excess H_2_SO_4_ to [DMBA][HSO_4_] revealed that excess acid did not react with either species in the protic IL but instead forms a mixture of the IL and sulfuric acid that exhibits decreasing *H*_0_ values with increasing acid–base ratio (Figure S2), consistent with previous work.^42^ Finally, we investigated the effect of the IL molar density on the observed Hammett acidity trends by considering the number of acidic protons in solution per unit volume of protic IL (Figure S3). This revealed that cation substitution is found to have a significant effect on measured *H*_0_; *H*_0_ values of [BA][HSO_4_], [HA][HSO_4_], and [MBA][HSO_4_] correspond to that of [DMBA][HSO_4_] containing 32%, 31%, and 11% excess sulfuric acid, and we concluded that density differences do not play a significant role in the observed acidity trends.

To rationalize the role of cation structure and the addition of water on the structure and acidity of our four alkylammonium [HSO_4_] protic ILs, we performed a series of classical and *ab initio* MD (CMD and AIMD) simulations and select quantum chemical (QM) calculations. For CMD simulations, we adopted the General Amber Force Field (GAFF) to model the atomic interactions.^43^ The application of GAFF to aqueous ILs has been previously investigated by Sprenger et al.^44^ Details of the computational procedures applied and validation of the model are provided in the Supporting Information along with computed and experimental densities (Table S2 and Figure S4). Analysis of cation(N)–anion(S) (Figure S5) and ion(N or S)–water(O) (Figure S6) pair radial distribution functions (prdfs) from the CMD simulations reveals that the local cation–anion structuring is dictated by the degree of substitution at the N-site of each cation, i.e., a higher number of N–H hydrogen bond donor sites leads to a greater number of anions directly interacting with the respective cation. Moreover, we find that water molecules form direct interactions with both cationic and anionic species. This leads to a systematic reduction in the intensity of ion–ion interactions relative to the anhydrous systems while the main structuring of the individual protic ILs remains intact with water molecules intercalated in the ionic network and unable to form bulk water-like structure, consistent with recent findings from neutron scattering experiments on pyridinium hydrogen sulfate, [Hpy][HSO_4_], doped with ∼15 wt % water.^45^

The effect on structuring and corresponding chemical properties has been previously investigated for several ILs on addition of metal salt dopants^46,47^ or water at various concentrations.^48−51^ Specifically, addition of metal dopants revealed unsymmetrical anion–metal cation clusters, which directly impacts the transport properties of the metal cations.^47^ Whereas, addition of water leads to the formation of anion–water, cation–water, and water–water clusters and may facilitate the percolation of water throughout the ILs dependent on water concentration.^49^ For our PILs doped with 20 wt % ILs, we determined the probability distribution of the average number of water neighbors per water molecule (Figure S7a) and found ∼2–3 neighbors compared to bulk water, approximately 4–5 neighboring water molecules. This indicates the formation of different water nanostructures in the PILs. To further investigate this, we computed water–water clusters (Figures S7b and S8) and identified both small clusters, representative of strong water–ion interactions, and larger clusters (linked water–water interactions) that percolate the length of the simulation cells (Figures S7c and S8). The latter percolating water networks are important for enabling long-range proton transfer and are being actively investigated for alkylammonium [HSO_4_]^−^ protic ILs with varying water content.

Next, we investigated the formation of [HSO_4_]^−^ anion–anion chains and discrete anionic clusters, connected via hydrogen bonds, which have been observed in the gas phase, in aqueous H_2_SO_4_ solution and in IL crystal structures. We assess the formation of anion–anion aggregates by first considering the anion(S)–anion(s) prdfs (Figures 1C and 1D). For each of our alkylammonium [HSO_4_]^−^ protic ILs, we find a first solvation maxima at ∼4.6–4.7 Å corresponding to anion–anion aggregation. This interaction is present but diminished with the introduction of 20 wt % water. Next, we computed anion–anion aggregate size distributions (Figure S9), average number of anion–anion domains (Figure 1E), and average number of anion neighbors around an [HSO_4_]^−^ anion (Figure 1F). For both anhydrous and doped protic ILs, we find that the degree of substitution on the cation directly influences the average number of anion–anion domains and average number of direct anion neighbors. For example, primary substituted [BA][HSO_4_] doped with 20 wt % water exhibits 40 anion–anion domains increasing to 132 domains for tertiary substituted [DMBA][HSO_4_] doped with 20 wt % water. Moreover, from the average number of direct neighbors of protic ILs doped with 20 wt % water, we find preferential formation of anion dimers for [DMBA][HSO_4_], a mixture of anion dimers and trimers for [MBA][HSO_4_], and predominantly anion trimers for [BA][HSO_4_] and [HA][HSO_4_]. Based on these initial findings, we hypothesize that an increased propensity for formation of higher concentration [HSO_4_]^−^ anion aggregates (trimeric over dimeric) is a key structural feature leading to the increased acidity of primary substituted alkylammonium [HSO_4_] protic ILs over their secondary and tertiary counterparts.

Next, we performed AIMD simulations on each of the four alkylammonium [HSO_4_]^−^ protic ILs doped with 20 wt % water. This was to complement our CMD simulation results and investigate the propensity for spontaneous acid dissociation of [HSO_4_]^−^ anions in our systems. To explore each of the two possible proton dissociation options (i.e., [HSO_4_]^−^ to water and [HSO_4_]^−^ to [HSO_4_]^−^) and one proton transfer option (i.e., [H_3_O]^+^ to water), we plotted a series of combined distribution functions for each system. In our 100 ps simulations, we observed spontaneous proton dissociation from [HSO_4_]^−^ to water only for the primary amines [BA][HSO_4_] and [HA][HSO_4_] (Figure 2). In these systems, the dissociation was preceded by the formation of an anion trimer motif. Additionally, we found incomplete proton dissociation for [MBA][HSO_4_] and no proton dissociation for [DMBA][HSO_4_]. We also observed proton transfer between adjacent water molecules following the initial formation of an [H_3_O]^+^ cation (Figure 3) and evidence of partial proton transfer between [HSO_4_]^−^ anions only for the primary amines (Figure S10).

**Figure 2:**
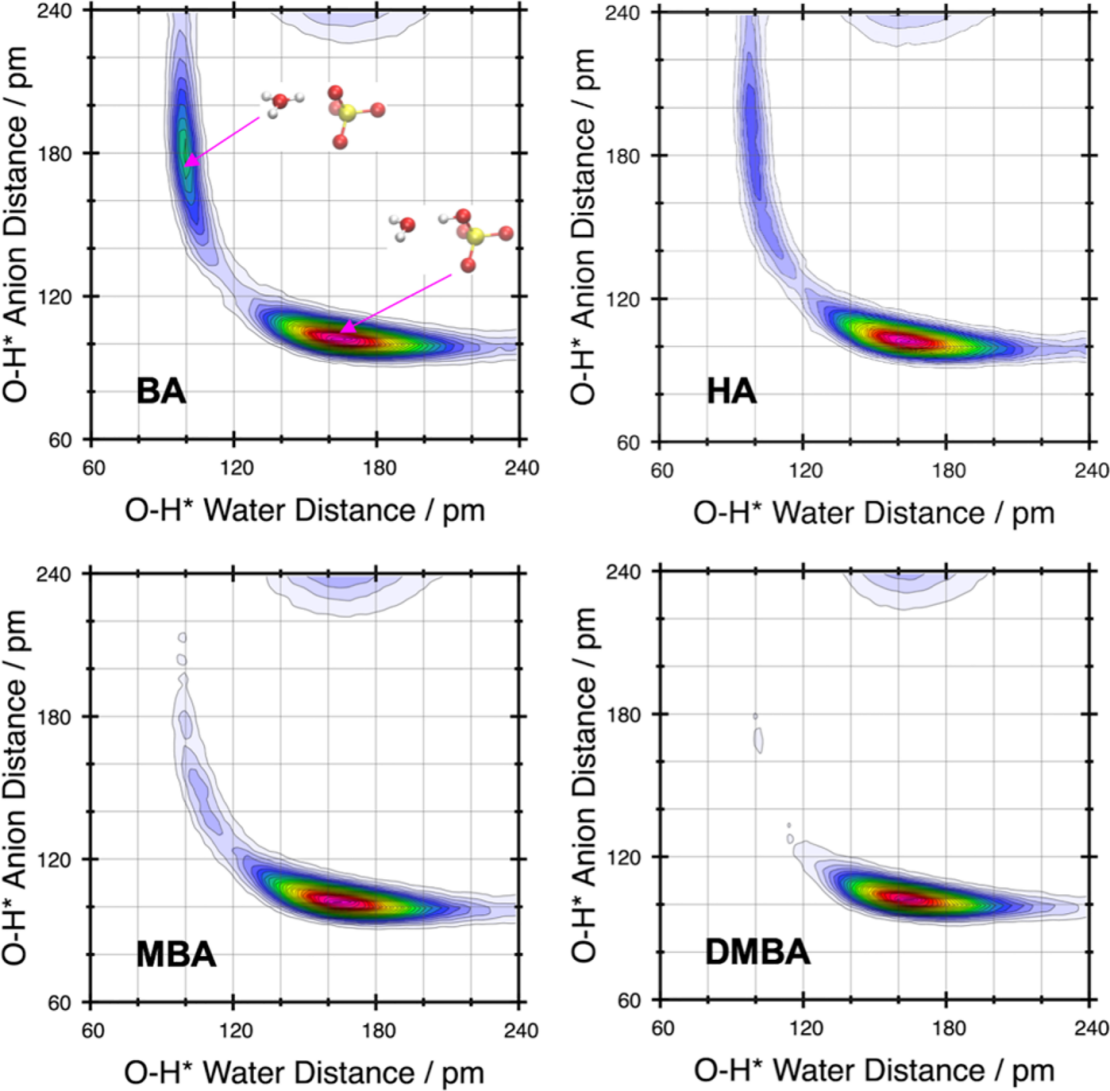
Combined radial distribution functions of the water O···H* vs anion O···H* distance, where H* originates from the acidic [HSO_4_]^−^ anion.

**Figure 3:**
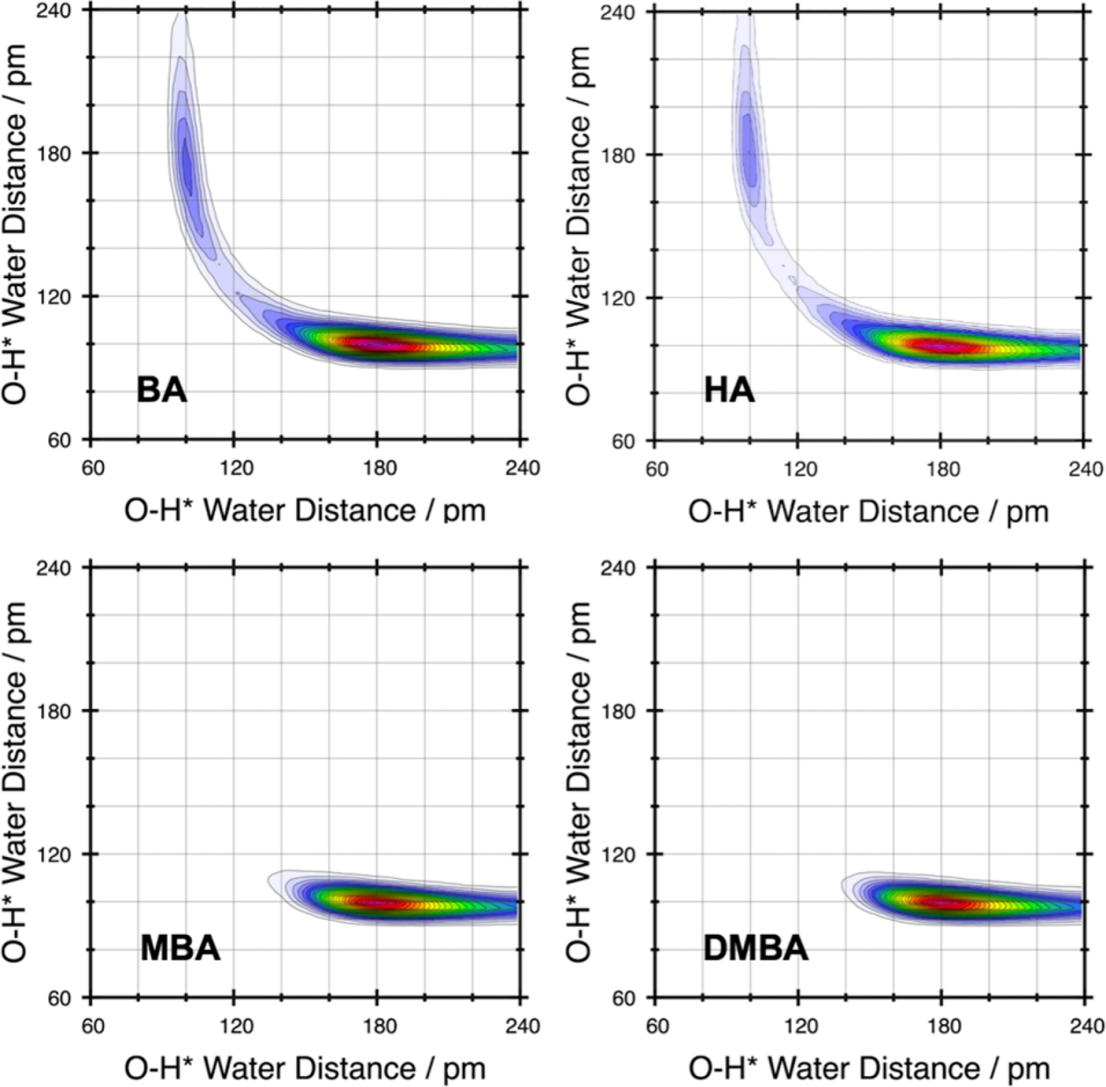
Combined RDFs of water O···H* vs water O···H* distance, where H* represents the labile proton on a water molecule or hydronium [H_3_O]^+^ cation.

Accordingly, the propensity for proton dissociation and transfer from AIMD simulations, [BA][HSO_4_] ≥ [HA][HSO_4_] > [MBA][HSO_4_] > [DMBA][HSO_4_], agrees with our measured experimental acidities. Moreover, CMD and AIMD simulations indicate that the formation of trimeric [HSO_4_]^−^ anionic clusters is a key feature of increased acidity of primary over secondary and tertiary substituted based alkylammonium [HSO_4_]^−^ protic ILs leading to increased acidity. However, there is the possibility that for the more highly substituted [MBA][HSO_4_] and [DMBA][HSO_4_] protic ILs dynamic processes leading to proton dissociation and transfer may be slower than for primary substituted [BA][HSO_4_] ≥ [HA][HSO_4_], which the reorientation dynamics of water and [HSO_4_]^−^ would be hampered by the lower hydrogen bonding abilities of the cations, similar to that observed for water reorientation in hydrophobic environments.^52,53^ Consequently, both proton dissociation and transfer for [MBA][HSO_4_] and [DMBA][HSO_4_] likely occur (a) on longer time scales or (b) are thermodynamically controlled. Both avenues of thought are actively being pursued.

Finally, we performed a series of DFT calculations containing cation–anion ion pair dimers and trimers for each of the alkylammonium [HSO_4_]^−^ protic ILs, anhydrous and doped with ∼20 wt % water. Initial geometries were constructed based on selected anion structural motifs (Figure S11); waters were included to best reflect our CMD and AIMD results, i.e., 3 water molecules per [HSO_4_]^−^ anion. Previous investigations of ammonium [HSO_4_]^−^ aerosols have indicated that lowest energy conformers are obtained where cation–anion hydrogen bonds are maximized.^54^Figure 4 shows key structural features pertaining to the dimer structures, including explicit inclusion water molecules. Similar IL structural arrangements were found for anhydrous dimers (Figure S12 and Table S3), for which we find the cyclic dimer motif (Figure 4A) to be most energetically favored by 15–25 kJ mol^–1^ over a chain dimer (Figure 4B) for all systems. The cyclic motif is further stabilized via anion–anion (AEHB) antielectrostatic hydrogen bonds,^55,56^ which increase in strength with more substituted cations as revealed via QTAIM and NBO analysis (Figure S13). On addition of 6 water molecules, we find water molecules preferentially interact with the cations via hydrogen bonds (i.e., N^+^-H---O) followed by interaction with [HSO_4_]^−^ anions. Consequently, the lowest energy structures for primary [BA] and [HA] cations retain the cyclic dimer (Figure 4A and Table S4). Moreover, increased substitution in secondary and tertiary cations (i.e., [MBA] and [DMBA]) results in preferential water interactions with the [HSO_4_]^−^ anions, facilitating competition between cyclic and chain dimer structures (Figure 4B). This is consistent with the CMD and AIMD results, which indicate the decrease in the level of anion–anion cluster formation with increased substitution on the cation. Furthermore, we find that the possibility of waters located between the anion (Figure 4C), forming anion–water–anion chains, to be less energetically favorable. Finally, we explored the potential for proton dissociation from [HSO_4_]^−^ to water for the cyclic dimer structures. This was only found to be slightly energetically favorable via the formation of small clusters of 4–5 water molecules located at the OH site on a [HSO_4_]^−^ anion (Figure 4D), which is inconsistent with the structuring from MD simulations where each anion is surrounded by 2.6 water molecules on average.

**Figure 4:**
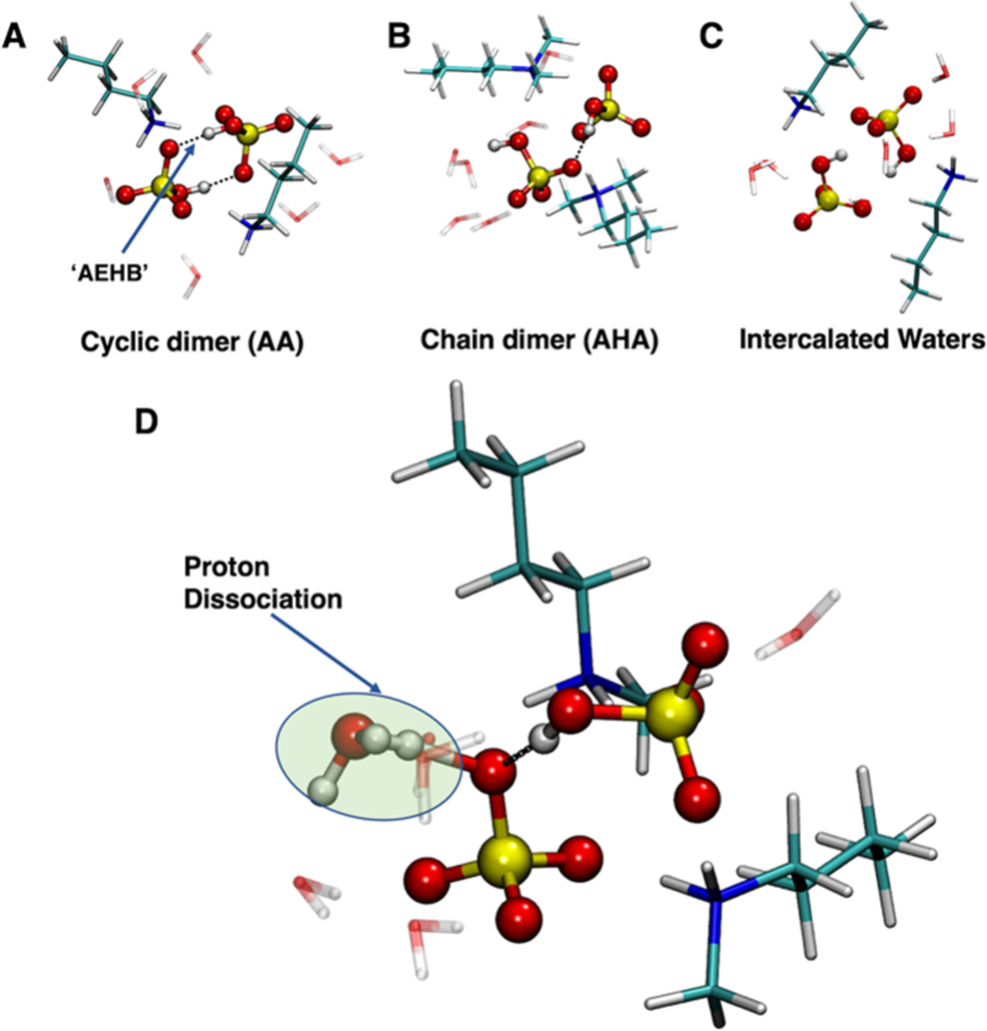
Selected features for ion pair dimers containing ∼20 wt % water (3 H_2_O molecules per ion pair). (A) Cyclic chain (AA) structure, (B) a chain dimer (AHA) with 4 water molecules at the open OH site, (C) two intercalated (IC) water molecules between the anion species, and (D) proton transfer (PT) from [HSO_4_]^−^ to a water molecule. PT requires a perturbed system, where at least 4 water molecules are present at the open OH site of a [HSO_4_]^−^ anion.

For protic IL trimers, with explicit inclusion of 9 water molecules, we find proton dissociation to be energetically favorable for [BA][HSO_4_], [HA][HSO_4_], and [DMBA][HSO_4_]. However, for [MBA][HSO_4_] we find proton dissociation to be governed by the entropic contribution to the free energy. Consequently, proton dissociation will readily occur at higher temperatures. Figure 5 shows the lowest energy conformers, with and without proton dissociation, for each of the alkylammonium [HSO_4_]^−^ protic ILs (energies are reported in Table S5). Proton dissociation, from [HSO_4_]^−^ to a water molecule to form [SO_4_]^2–^ and [H_3_O]^+^, is found to be dependent on the structure of the anion trimer motif (i.e., a double-chain trimer arrangement (Figure 5B). For [DMBA][HSO_4_] we find a chain-cyclic trimer anionic motif is preferred over the cyclic trimer motif found for the three other investigated protic ILs. This finding closely aligns with the anion cluster sizes from CMD simulations. Moreover, for [DMBA][HSO_4_] we find proton dissociation to be highly energetically favorable (∼20 kJ mol^–1^) on adopting a double chain trimer motif (Figure 5A). This is a surprising result and together with observations from the dimer calculations suggests that proton dissociation may be significantly altered by perturbing the protic IL structure by varying water concentrations, in agreement with experimental *H*_0_ values found for [DMBA][HSO_4_] with 10–40 wt % water.^10,57^

**Figure 5:**
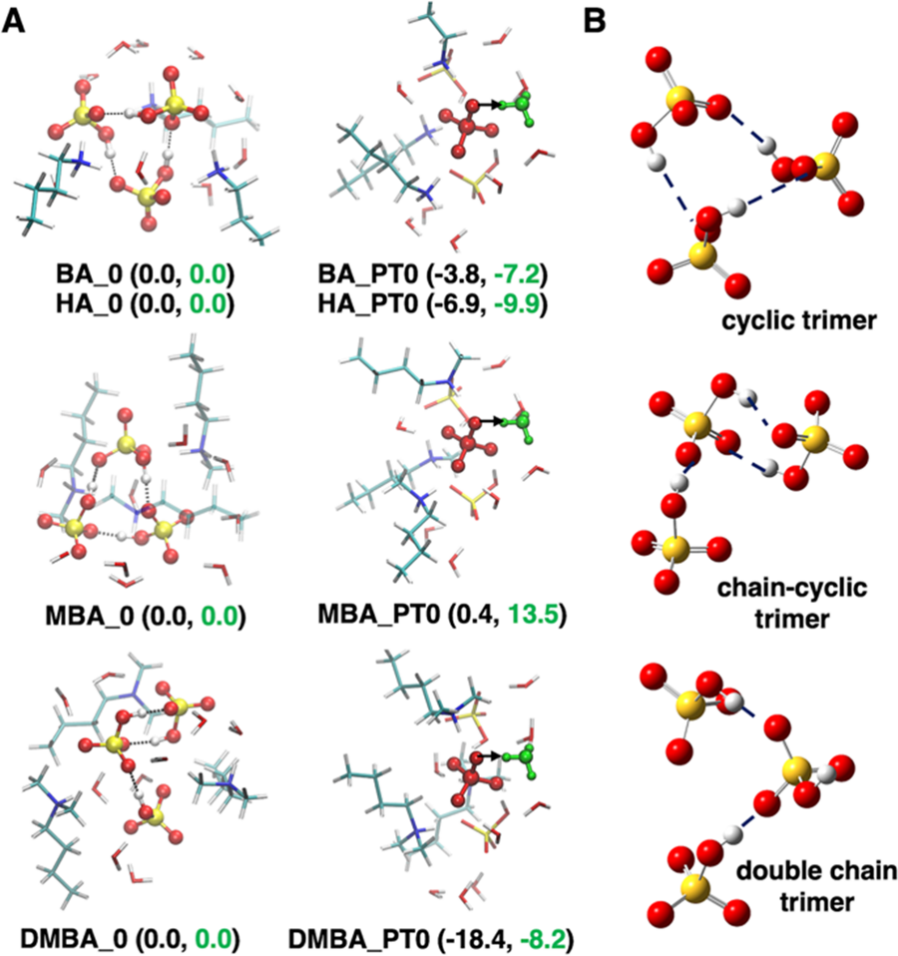
(A) Selected conformers of the ion pair trimers containing ∼20 wt % water (3 H_2_O molecules per ion pair). They are labeled according to whether proton transfer has (X_PT0) or has not (X_0) occurred. Conformer energies are reported in kJ mol^–1^. Δ*E*_ZPE_ and Δ*G* are colored black and green. (B) Important anion trimer structural motifs.

In summary, we have shown a direct correlation between the degree of substitution on the cation and the acidity of alkylammonium [HSO_4_] protic ILs doped with 20 wt % water. From classical and *ab initio* MD simulations, we have identified that proton dissociation (and higher acidity) is more readily achieved with an increased number of N–H hydrogen bond donor sites on the cation, which also more readily facilitates the formation of anionic trimeric clusters. Similarly, from DFT calculations, we have identified that the proton dissociation from [HSO_4_]^−^ anions to water proceeds via a trimeric anion formation and can be further modulated by varying the number of waters at the dissociating proton site. We hope that these findings will drive further research in developing solvent environments used in acid based catalysis research and lignocellulosic biomass treatment where solvent acidity plays a crucial role in biomass fractionation.

## Supporting Information Available

Full details of experimental materials and procedures and classical and ab initio molecular dynamics simulations and DFT calculations; additional figures outlining auxiliary acidity measurements; computed and experimental densities, pair radial distribution functions, anion–anion and water–water aggregates of PILs doped with 20 wt % water from classical MD simulations; combined radial distribution functions outlining anion–anion proton transfer from *ab initio* MD simulations and figures and tables detailing the structures and energetics of dimeric and trimeric systems from DFT calculations (PDF)
