# Supplementary material for: Exploiting Cation Structure and Water Content in Modulating the Acidity of Ammonium Hydrogen Sulfate Protic Ionic Liquids
Source: J Phys Chem Lett. 2024 Feb 22;15(9):2311–8. doi: 10.1021/acs.jpclett.3c03583 (PMC10926163; doi:10.1021/acs.jpclett.3c03583)
Supplement: Supplementary file 1 — jz3c03583_si_001.pdf [file jz3c03583_si_001.pdf]

Exploiting Cation Structure and Water Content in Modulating the Acidity of Ammonium Hydrogen Sulfate Protic Ionic Liquids

Supporting Information

Anton E. J. Firth[a], *Pedro Y. S. Nakasu*[a], *Jason P. Hallett*[a*] *and Richard P. Matthews*[a,b]*

[a] Department of Chemical Engineering, Imperial College London, London, SW7 2AZ, UK.

[b] Department of Bioscience, School of Health, Sports and Bioscience, University of East London, Stratford, London, E15 4LZ, UK.

Corresponding Authors

* j.hallett@imperial.ac.uk; rmatthews3@uel.ac.uk

A. Materials and Experimental Methods

Starting Materials

N-methyl-n-butylamine (>98.1%) and 5M sulfuric acid were purchased from VWR, n-butylamine (>99.5%), n-hexylamine (>99.0%), 4-nitroaniline (>99%), and anhydrous dichloromethane (>99.8%) were purchased from Sigma Aldrich, and *N*,*N*-dimethyl-n-butylamine (>98.0%) was purchased from Tokyo Chemicals Industry. All the above were used as received.

IL synthesis and NMR Characterisation

Using a dropping funnel, sulfuric acid was added dropwise to a round bottomed flask containing the corresponding amine, magnetically stirred and chilled to 0°C using an ice bath. IL water content was reduced to below 20% by rotary evaporation as measured using a V20 Volumetric Karl-Fischer Titrator (Mettler-Toledo), standardised using Hydranal water standards. Acid-base ratios were determined by titration by 0.1M NaOH, using a G20S Compact Titrator (Mettler-Toledo), standardised using potassium hydrogen phthalate purchased from Sigma Aldrich. Acid-base ratios are expressed on a molar basis.

N,N-Dimethyl-N-n-ButylAmmonium Hydrogen Sulfate ([DMBA][HSO_4]):
^{1}H NMR: δ_H (400 MHz, DMSO-d_6)/ppm: 9.23 (s, 1H, N-*H*), 5.65 (s, br, H_2O, HSO_4), 2.98 (t, J = 8.4, 2H, N-CH_2), 2.72 (s, 6H, N-(CH_3)$_2$), 1.54 (q, J = 7.6, 2H, N-CH_2-CH_2), 1.25 (m, 2H, N-CH_2-CH_2-CH_2), 0.85 (t, J = 7.8, 3H, N-CH_2-CH_2-CH_2-CH_3).
^{13}C NMR δC (101 MHz, DMSO-d_6)/ppm: 57.00 (N-(CH_3)$_2$), 42.76 (N-CH_2), 26.23 (N-CH_2-CH_2), 19.72 (N-CH_2-CH_2-CH_2), 14.01 (N-CH_2-CH_2-CH_2-CH_3).

N-Methyl-N-n-ButylAmmonium Hydrogen Sulfate ([MBA][HSO_4]):
^{1}H NMR: δ_H (400 MHz, DMSO-d_6)/ppm: 8.31 (s, 2H, N-H_2), 6.41 (s, br, H_2O, HSO_4), 2.98 (m, 2H, N-CH_2), 2.55 (t, J = 5.5, 3H, N-CH_3), 1.54 (m, 2H, N-CH_2-CH_2), 1.31 (h, J = 7.4, 2H, N-CH_2-CH_2-CH_2), 0.89 (t, J = 7.4, 3H, N-CH_2-CH_2-CH_2-CH_3).
^{13}C NMR δC (101 MHz, DMSO-d_6)/ppm: 48.64 (N-CH_3), 33.05 (N-CH_2), 27.81 (N-CH_2-CH_2), 19.63 (N-CH_2-CH_2-CH_2), 13.96 (N-CH_2-CH_2-CH_2-CH_3).

HexylAmmonium Hydrogen Sulfate ([HA][HSO_4]):
^{1}H NMR: δ_H (400 MHz, DMSO-d_6)/ppm: 7.67 (s, 3H, N-H_3), 4.19 (s, br, H_2O, HSO_4), 2.75 (m, 2H, N-CH_2), 1.51 (m, 2H, N-CH_2-CH_2-CH_2-CH_2-CH_2), 1.27 (m, 6H, N-CH_2-CH_2-CH_2 -CH_2), 0.86 (m, 3H, N-CH_2-CH_2-CH_2-CH_2-CH_2-CH_3).
^{13}C NMR δC (101 MHz, DMSO-d_6)/ppm: 39.41 (N-CH_2), 31.17 (N-CH_2-CH_2), 27.33 (N-CH_2-CH_2-CH_2), 25.90 (N-CH_2-CH_2-CH_2-CH_2), 22.33 (N-CH_2-CH_2-CH_2-CH_2-CH_2), 14.31 (N-CH_2-CH_2-CH_2-CH_2-CH_2-CH_3).

ButylAmmonium Hydrogen Sulfate ([BA][HSO_4]):
^{1}H NMR: δ_H (400 MHz, DMSO-d_6)/ppm: 7.68 (s, 3H, N-H_3), 4.45 (s, br, H_2O, HSO_4), 2.77 (m, 2H, N-CH_2), 1.52 (m, 2H, N-CH_2-CH_2), 1.32 (m, 2H, N-CH_2-CH_2-CH_2), 0.88 (t, J = 7.3, 3H, N-CH_2-CH_2-CH_2-CH_3).
^{13}C NMR δC (101 MHz, DMSO-d_6)/ppm: 39.12 (N-CH_2), 29.43 (N-CH_2-CH_2), 19.53 (N-CH_2-CH_2-CH_2), 13.94 (N-CH_2-CH_2-CH_2-CH_3).

Hammett Acidity Measurements

IL Hammett acidity was measured using UV-Vis, combining the Beer-Lambert law (**Equation 1**) and a modified form of the Henderson-Hasselbalch equation (**Equation 2**) as in the work of Grasvik et al.[1]

$$A = \varepsilon \times c \times l \quad (1)$$

Where *A* is absorbance, ε is absorptivity, *c* is concentration, *l* is path length.

$$H_0 = pK_{BH^+} - log\left(\frac{[\varepsilon_0] - [\varepsilon]}{[\varepsilon]}\right) \quad (2)$$

Where H_0 is Hammett acidity, pK_{BH^+} is the basicity constant of the Hammett base, ε_0 is the absorptivity of the fully unprotonated Hammett base, and ε is the absorptivity of the partially protonated Hammett base.

The Hammett base used in this work was 4-nitroaniline. This base has been previously reported to be suitable for the range of Hammett acidities investigated.[2] The pK_{BH+} value of 4-nitroaniline is 1.00, obtained from literature.[1, 3] The absorptivity of fully unprotonated 4-nitroaniline (ε_0) was determined experimentally to be 16,600 $M^{-1}cm^{-1}$, which is consistent with values previously reported by Grasvik et al.[1] This was done by measuring the UV-Vis absorbance at 380 nm of 5 different concentrations of the Hammett base in anhydrous dichloromethane, and measuring the gradient of the *A* vs. *cl* graph. All other absorptivity values (ε) were determined by measuring the UV-Vis absorbance of the unprotonated peak of 4-nitroaniline (380 nm) for 5 different concentrations of the Hammett base in the relevant ionic liquid and measuring the gradient of the *A* vs. *cl* graph. All Hammett acidity measurements were repeated twice, i.e. preparing new stock solutions of 4-nitroaniline in DCM and ionic liquid, which were then diluted to the desired concentrations. All UV-Vis measurements were performed using a Perkin Elmer Lambda 650, with solutions pipetted into sealed UV-clear quartz cuvettes with a path length of 0.5 cm.

Density Measurements

Aqueous IL solution densities were measured using a Mettler-Toledo DM40 density meter, between 0 °C and 50 °C. Solutions were sonicated for 20 minutes before measurements to remove any bubbles and dissolved air. The cell condition was verified before each use by measuring the density of purified water at 20 °C (0.99821 g/cm^3), and ensuring it was within the tolerance (0.00008 g/cm^3).

B. Computational Methods

Classical Molecular Dynamics

Classical MD simulations were carried out using the Amber16 program.[4] Structural and dynamical analysis of each trajectory was accomplished using the open-source programs TRAVIS,[5, 6] AGGREGATES[7] and CPPTRAJ.[8] The protic ionic liquids (PILs) – [BA][HSO_4], [HA][HSO_4], [MBA][HSO_4] and [DMBA][HSO_4] – were modelled using the General Amber force field (GAFF)[9] and the four-site TIP4P-EW[10] water model was used in simulations that included water molecules. The GAFF potential has been recently applied to similar PILs systems,[11] and has been shown to be able to predict thermodynamic and transport properties of several ILs, both pure and when combined with water (i.e. doped or aqueous IL).[12] In addition, GAFF is recommended where limited experimental data is available.[12] Furthermore, Tenney et al. have previously employed GAFF for the investigation of ILs and have noted that in general the initial GAFF parameterised assigned require very minor refinements.[13] Consequently, we employ the GAFF parameters as assigned. Next, partial atomic charges for each of the PIL species were assigned using the RESP[14] (restrained electrostatic potential) method at the B3LYP/6-311++g(d,p) level of theory. This approach has been previously shown to yield reliable charges for organic molecules[15] and ILs.[13] As in the literature the charges of the PIL species were scaled by 0.8 to model the average charge screening due to polarisation and improve solvent dynamics.[12, 13]

Each simulation box comprised of 400 cation-anion ion pairs. For simulations containing 20 wt% water, the number of water molecules added for each system is provided in **Table S1**. Initial simulation configurations were generated using the PACKMOL program.[16] Each simulation has been carried out using periodic boundary conditions and a 1 fs time step. The particle mesh Ewald (PME) method was employed for long-range electrostatics interactions and a 12 Å cut-off was included for non-bonded interactions.[17] The temperature and pressure were controlled using the Langevin thermostat (γ = 1.0 ps^{-1}) and Berendsen barostat (p = 1 atm, τ = 1000 fs).[18, 19] All bonds containing hydrogen atoms were constrained using SHAKE.[20]

To equilibrate each system, we followed a procedure adapted from our previous work on IL mixtures.[21] Following an initial minimisation (steepest descent – 1000 steps and conjugated gradient – 4000 steps), each simulation was subjected to the following steps. 1) A heating phase, where each system was gradually heated to 600 K over 5 ns in the NVT ensemble to remove possible energy hotspots. 2) A short (1 ns) equilibration was then carried out at 600 K in the NPT ensemble. 3) This was followed by a cooling phase, where each system was gradually cooled from 600 K to 300 K over 5ns (NVT). 4) A further 10 ns NPT simulation was carried out to obtain a consistent density. The average density for the last 5 ns of the each of the respective NPT simulations was then used to carry out 100 ns production simulations at 300 K in the NVT ensemble and the last 50 ns of each production simulation was used for analysis.

Additionally, each system was characterised by computing the density at different temperatures ranging from 273 K to 353 K in steps of 10 K (except for 300 K). Computed densities of the pure PILs and PILs doped with 20 wt% H_2O, together with experimentally measured densities for the doped PILs obtained during this study are reported in Figure S and Table S2. Moreover, for the pure PILs there is a lack of experimentally measured densities. Consequently, we have only included one data point for pure [BA][HSO_4]. Each of our simulated results were found to be within 2 % of those measured experimentally. Given the accuracy of these results and the great body of work carried out on ILs using the GAFF force field, we are confident with the selected model and the obtained results.

Table S1. The number of water molecules added to each classical molecular dynamics simulation to obtain a final concentration of 20 wt% H_2O. Each simulation contains 400 PIL ion pairs.

	**# H2O**
**[BA][HSO_4]**	1350
**[HA] [HSO_4]**	1506
**[MBA] [HSO_4]**	1428
**[DMBA] [HSO_4]**	1506
**TIP4P-EW (only)**	2500

Ab initio Molecular Dynamics

Ab initio molecular dynamics (AIMD) simulations were carried out using the QUICKSTEP[22] module within the CP2K[23] code. The BLYP[24, 25] functional was employed with the molecularly optimized double-ζ basis set (MOLOPT-DZVP-SR-GTH)[26] for each of the atoms and core electrons were represented by the corresponding BLYP Goedecker–Teter–Hutter[27-29] pseudopotentials. Dispersion interactions were accounted for using the DFT-D3 empirical dispersion correction.[30, 31] The resulting BLYP-D3 functional has been previously shown to produce good results for ionic liquid and hydrogen bonding systems.[32]

Initial simulation boxes were generated using PACKMOL[16] and contained 16 ion pairs of the corresponding protic IL and the commensurate number of water molecules. The systems were pre-equilibrated using following the method described above for classical MD simulations. For the AIMD equilibration phase, the plane wave cutoff was set at 200 Ry, together with multigrids number 5 (NGRID 5 and REL CUTOFF 30). Smoothing of the electron density was carried out using NN10 SMOOTH and its derivative (NN10).[22] The SCF convergence evaluation criteria was set to the default (10^{-5}) and the DIIS minimiser[23] was used to reach a faster orbital transformation via direct inversion in the iterative subspace. The maximum number of SCF iterations is 100 while a maximum of 10 iterations was performed for outer SCF loops. A further equilibration over 5 ps was performed using the keyword REGION MASSIVE, which means that for every single atom is thermostated individually for faster equilibration with a time constant of the thermostat chain of 50 fs. The temperature for this phase was set at 450 K to increase the sampling. Periodic boundary conditions were applied to avoid boundary effects.

A final production run was then carried out for each system in the NVT ensemble. For these runs, the density CUTOFF criterion was set to 350 Ry, together with NGRID 4 and REL CUTOFF 40. Moreover, the SCF convergence was set to 1.0E-6. A Nose-Hoover chain thermostat[33, 34] with a time constant 100 fs was used to obtain a target temperature of 300 K. Each simulation was subsequently run for 100 ps (using a 0.5 fs time step) and the last 90 ps were used for analysis.

Quantum Chemical Calculations

DFT calculations were carried out using Gaussian 16 (version c01).[35] Geometry optimisations were performed using the B3LYP[24, 36] functional and the 6–311++G(d,p) basis set. To account for dispersion interactions, Grimme's -D3[30, 31] dispersion correction with the Becke and Johnson dampening (BJ-dampening) function[37-39] was used. This combined method is referred to as B3LYP-D3BJ in the text. The combination of functional and basis set (B3LYP-D3BJ/6-311++g(d,p)) has been previously shown to provide good quality structures and energies for ionic liquid clusters. The Conductor-like Polarisable Continuum Model (CPCM) implicit solvent model was employed.[40, 41] The dielectric constant of ethanol was selected to represent the protic IL-water environment, based on the literature.[42] All structures have been fully optimised under no symmetry constraints and have been confirmed as minima using vibrational analysis. Optimisation convergence criteria were set to 10^{-9} on the density matrix and 10^{-7} on the energy matrix. The numerical grid was improved from the default to a pruned (optimised) grid of 99 radial shells and 590 angular points per shell. Vibrational frequencies and zero-point vibrational energy corrections (ZPE) were obtained within the harmonic approximation for each structure. Topological analysis of the electron density, within the quantum theory of atoms (QTAIM)[43] framework was carried out using AIMALL[44] and NBO analysis was carried out using NBO 6.0.[45, 46]

C. Supplementary Experimental Results

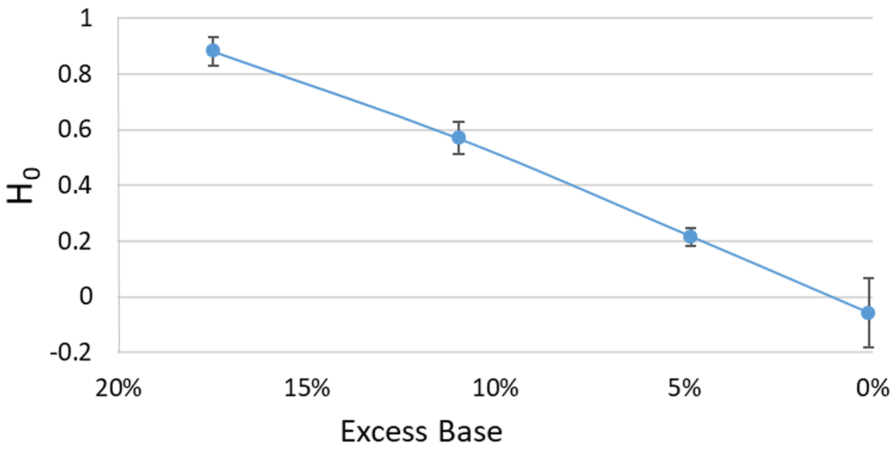

Figure S1: Variation of the Hammett acidity of [BA][HSO_4] with excess butylamine. All IL measurements were conducted using 20 wt % water. Excess base was measured on a molar basis.

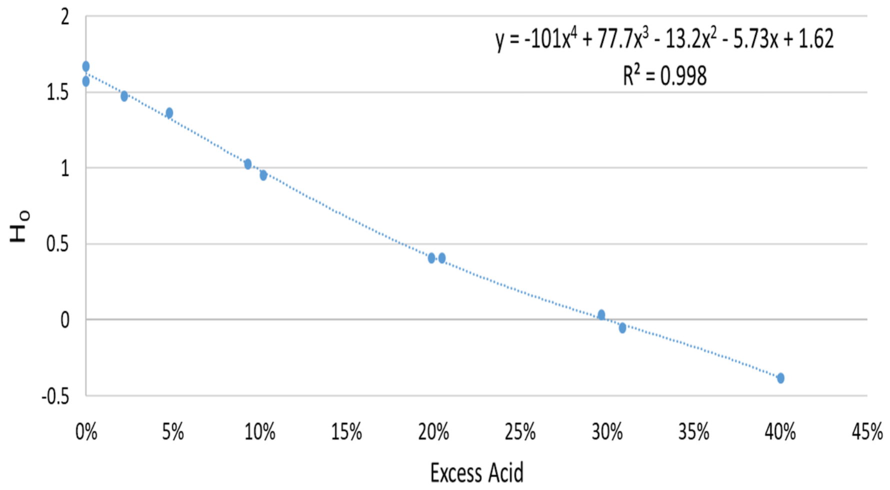

Figure S2: Variation of Hammett acidity of [DMBA][HSO_4] with excess sulfuric acid. All measurements were conducted using 20 wt % water. Excess acid was measured on a molar basis.

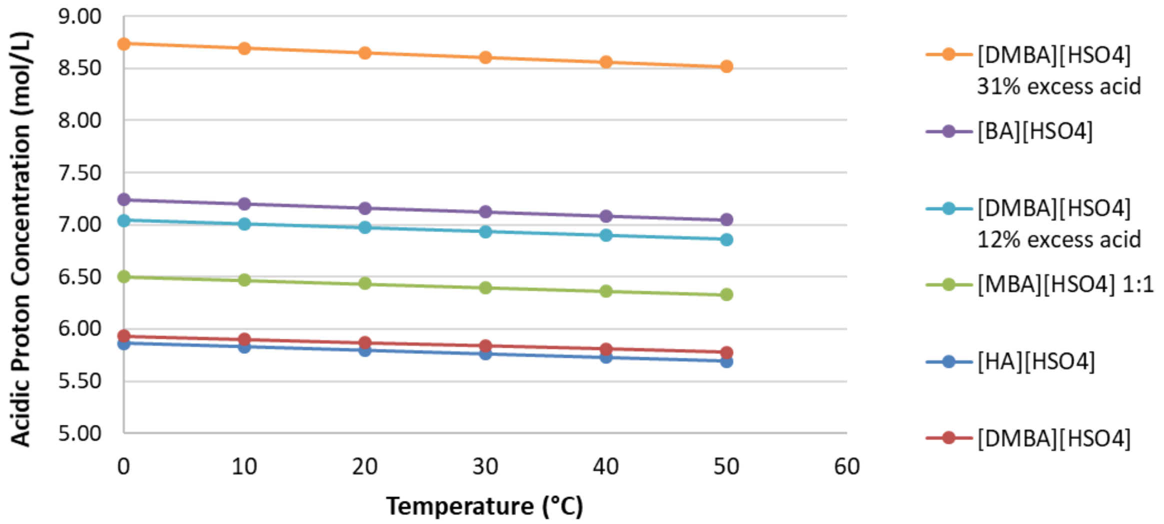

Figure S3: Variation of IL density with temperature. All IL measurements were conducted using 20 wt % water. Excess sulfuric acid was measured on a molar basis.

D. Supplementary Classical Molecular Dynamics Simulation Results

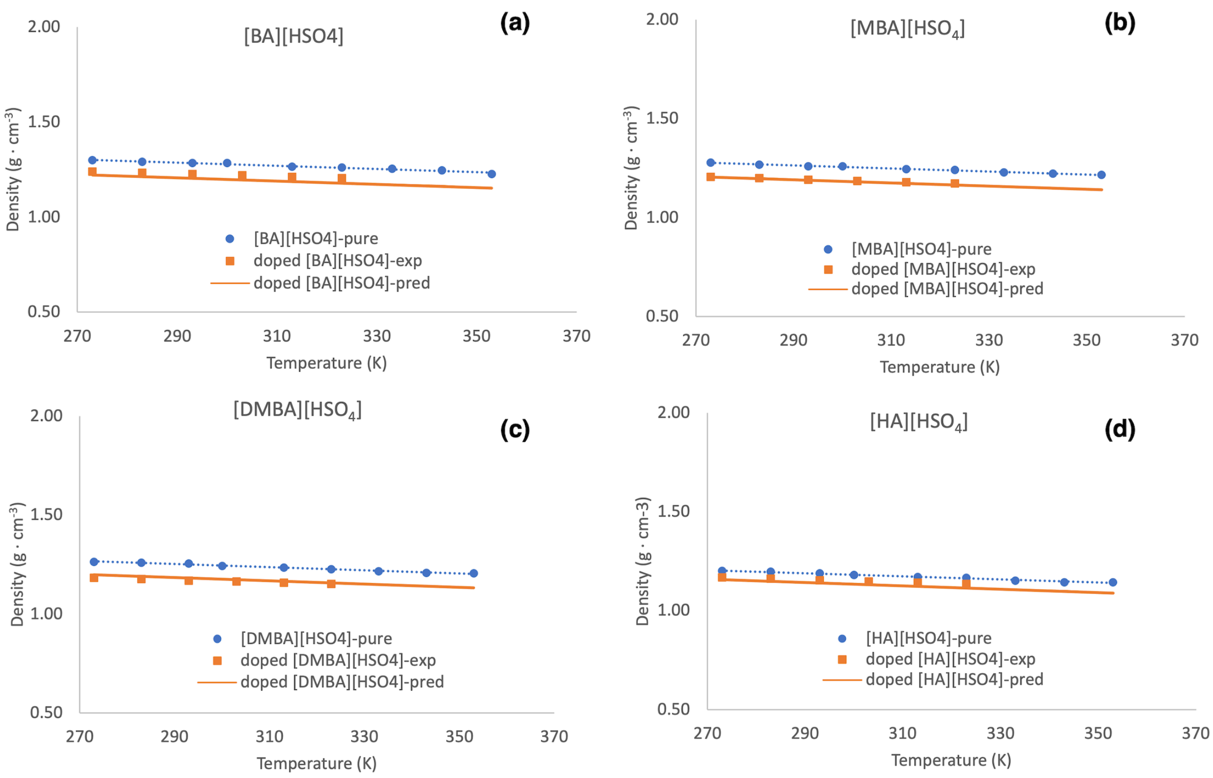

Figure S4: Experimental and computed Density vs temperature for pure PILs and PILs doped with 20 wt % H_2O. (a) [BA][HSO_4], (b) [MBA][HSO_4], (c) [DMBA][HSO_4] and (d) [HA][HSO_4]

Table S2. Computed and experimental densities for pure PILs and PILs doped with 20 wt % H2O determined in this work.

	Temp / K	Pure PILs Simulation	Pure PILs Experimental	Pure PILs % Difference	20 wt % H2O Simulation	20 wt % H2O Experimental	20 wt % H2O % Difference
[BA][HSO$_4$]	273	1.300			1.224	1.240	-1.3
	283	1.291			1.212	1.233	-1.7
	293	1.285			1.206	1.226	-1.6
	300	1.284	1.263[47] (298K)	1.7	1.195	1.220	-2.0
	313	1.266			1.184	1.213	-2.4
	323	1.262			1.182	1.207	-2.1
	333	1.255			1.172		
	343	1.247			1.165		
	353	1.228			1.151		
[MBA][HSO$_4$]	273	1.278			1.203	1.205	-0.2
	283	1.267			1.200	1.198	0.2
	293	1.259			1.189	1.192	-0.2
	300	1.259			1.181	1.185	-0.4
	313	1.245			1.176	1.179	-0.3
	323	1.240			1.166	1.173	-0.6
	333	1.229			1.154		
	343	1.221			1.147		
	353	1.216			1.141		
[DMBA][HSO$_4$]	273	1.264			1.200	1.182	1.5
	283	1.260			1.190	1.176	1.1
	293	1.255			1.183	1.169	1.1
	300	1.243			1.173	1.164	0.8
	313	1.236			1.168	1.158	0.9
	323	1.226			1.155	1.152	0.3
	333	1.216			1.149		
	343	1.208			1.141		
	353	1.206			1.134		
[HA][HSO$_4$]	273	1.201			1.158	1.169	-0.9
	283	1.197			1.149	1.162	-1.1
	293	1.189			1.141	1.155	-1.2
	300	1.182			1.134	1.148	-1.2
	313	1.171			1.123	1.141	-1.6
	323	1.167			1.113	1.134	-1.9
	333	1.152			1.105		
	343	1.144			1.101		
	353	1.143			1.088		

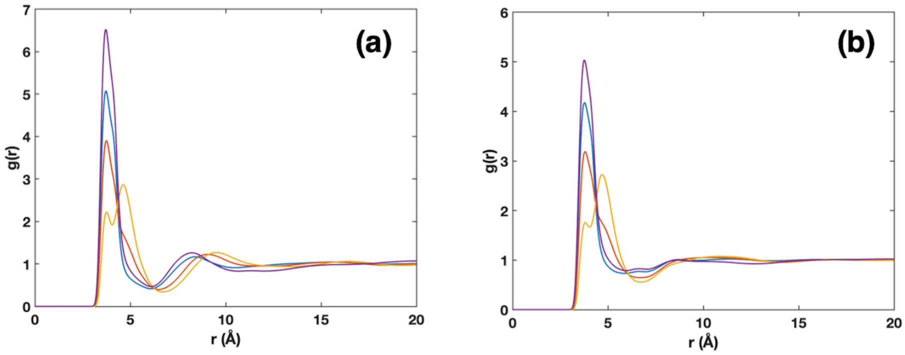

Figure S5: Cation(N)-anion(S) “ion-ion” pair radial distribution functions for (a) anhydrous protic ILs and (b) protic ILs doped with 20 wt % water. The RDFs for [BA][HSO_4] are shown in blue, [HA][HSO_4] in purple, [MBA][HSO_4] in orange and [DMBA][HSO_4] in yellow.

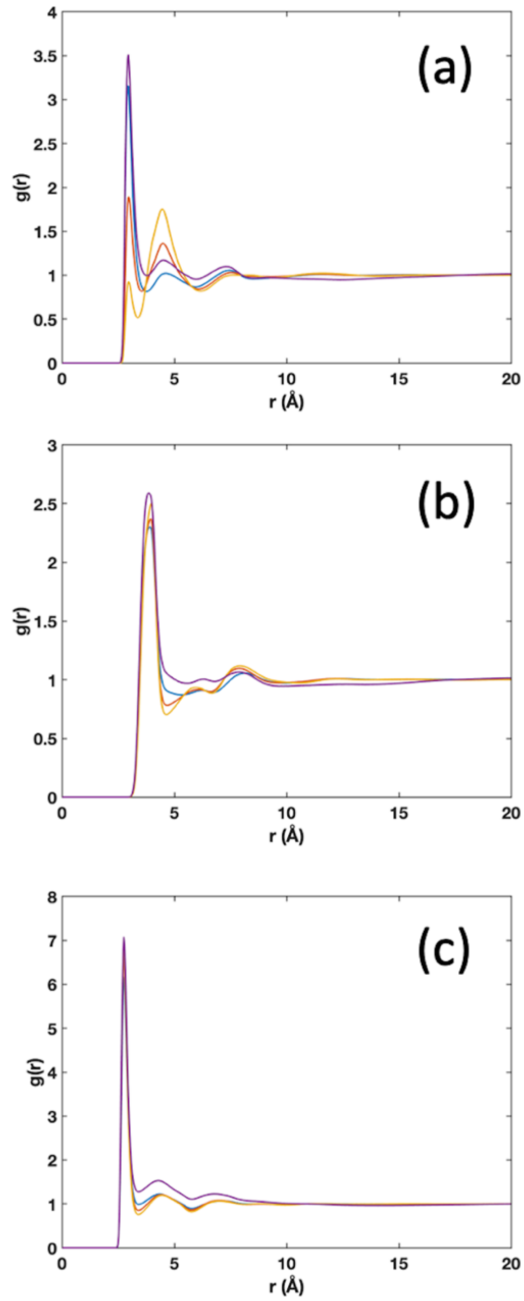

Figure S6: (a) Cation(N)-water(O), **(b)** anion(S)-water(O) and **(c)** water(O)-water(O) plot the respective "ion-water" pair radial distribution functions for the protic ILs containing 20 wt % water, obtained from the classical molecular dynamics simulations. The RDFs for [BA][HSO_4] are shown in blue, [HA][HSO_4] in purple, [MBA][HSO_4] in orange and [DMBA][HSO_4] in yellow.

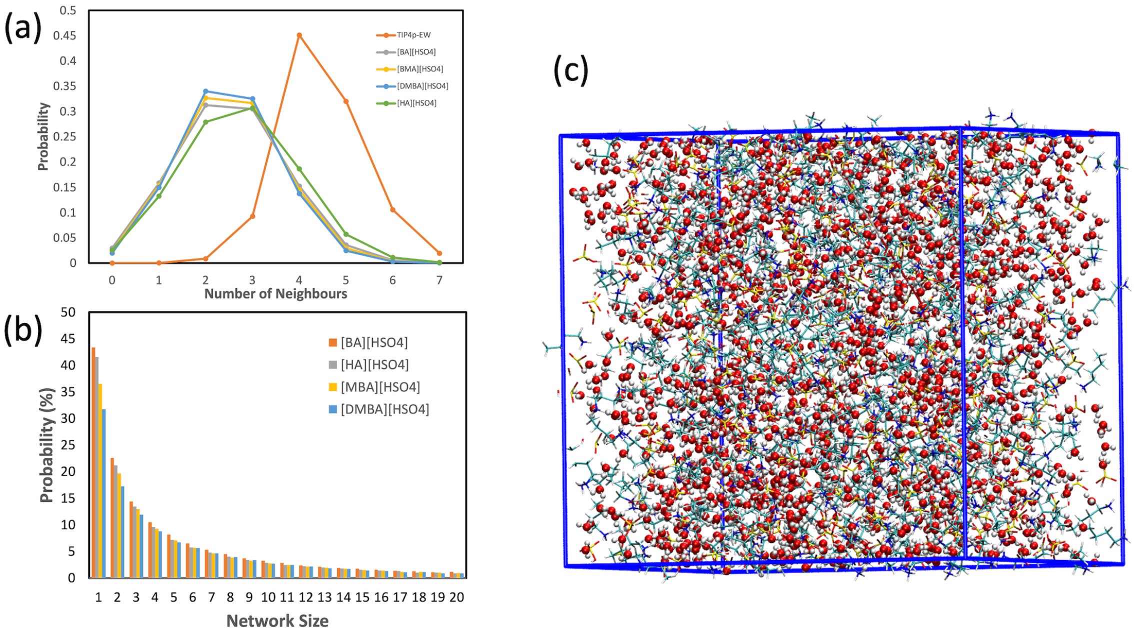

Figure S7: (a) Probability histogram of the number of water neighbours per water molecule for each of the PILs; [BA][HSO_4], [HA][HSO_4], [MBA][HSO_4] and [DMBA][HSO_4] doped with 20 wt % water, compared with a TIP4P-ew water simulation containing 2500 water molecules. (b) Histogram of the water-water network size up to 20 molecules for each of [BA][HSO_4], [HA][HSO_4], [MBA][HSO_4] and [DMBA][HSO_4] doped with 20 wt % water. (c) For comparison, a snapshot of the distribution of water taken from the simulation of [BA][HSO_4] doped with 20wt % water.

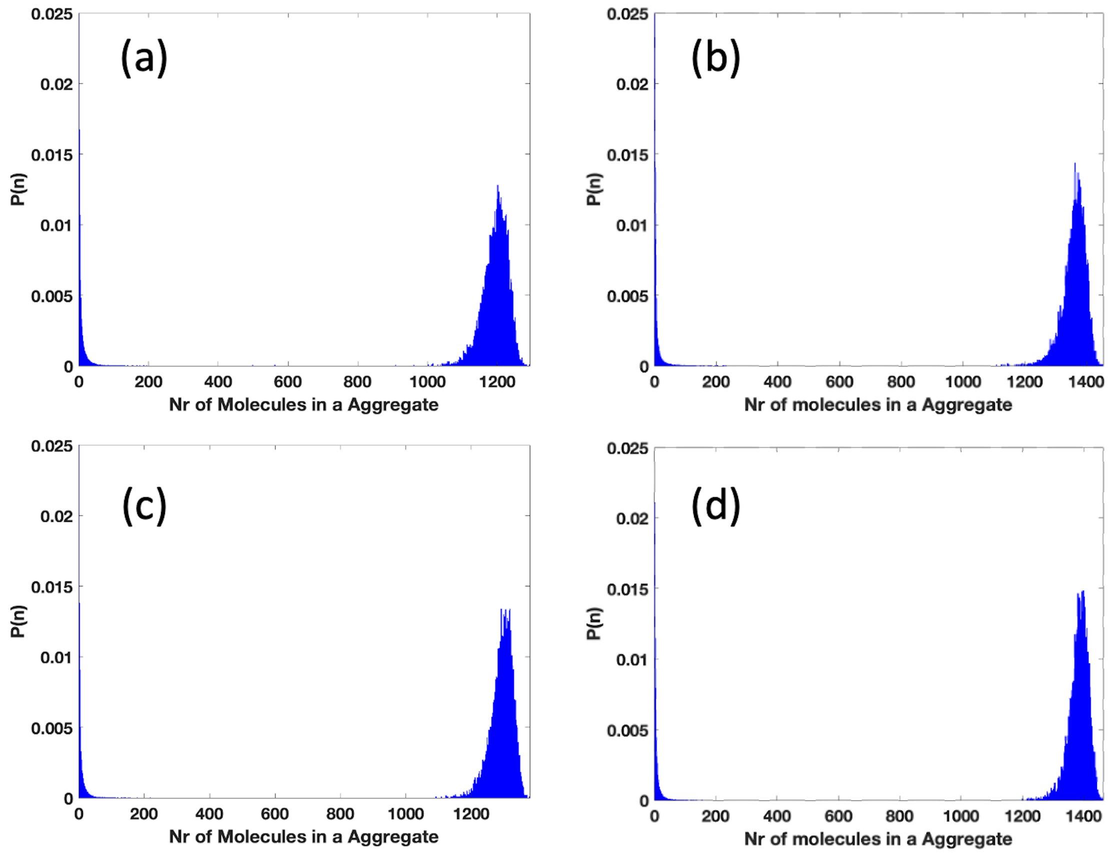

Figure S8: Size distributions of Water-Water aggregates for **(a)** [BA][HSO_4], **(b)** [HA][HSO_4], **(c)** [MBA][HSO_4] and **(d)** [DMBA][HSO_4], all doped with 20 wt % water, obtained from the classical molecular dynamics simulations. Size distribution data was computed using the AGGREGATES software, employing the corresponding O-O distance cut-off determined from the first minimum of the H_2O-H_2O radial distribution functions.

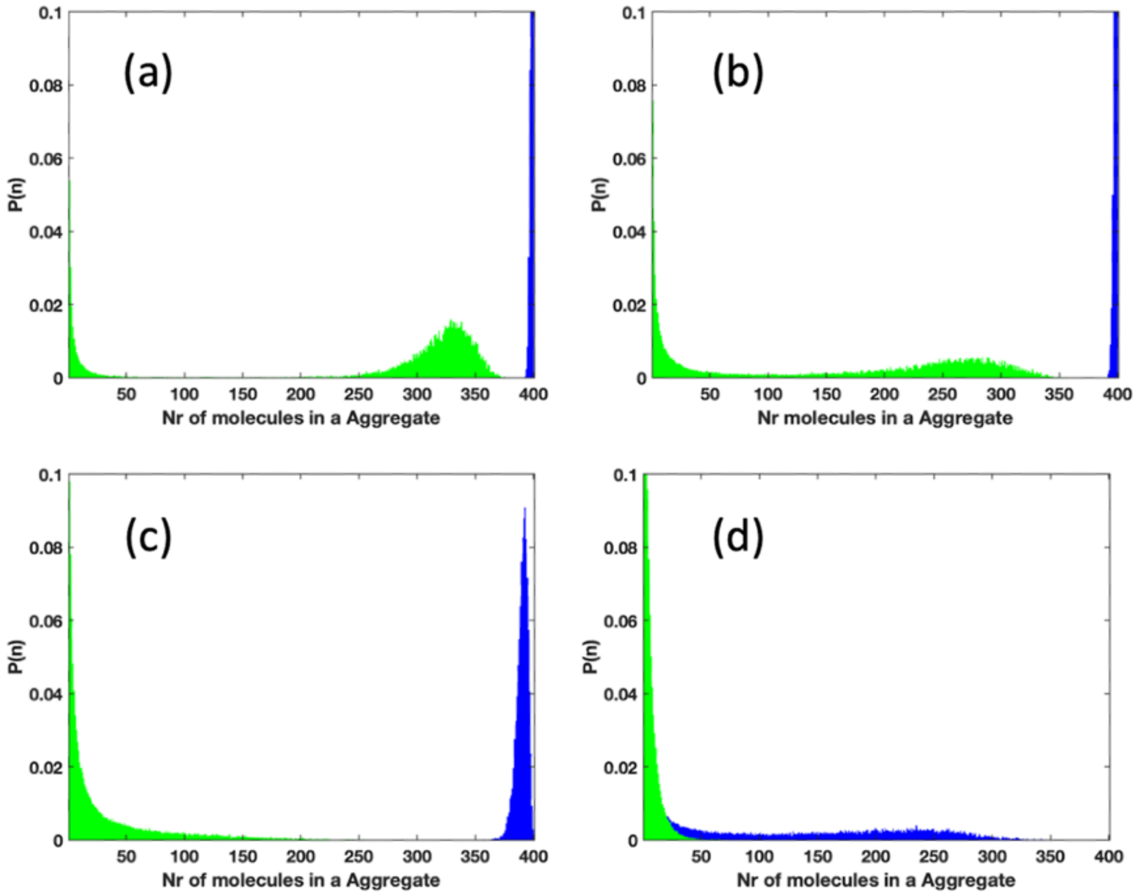

Figure S9: Size distributions of Anion-Anion aggregates for **(a)** [BA][HSO_4], **(b)** [HA][HSO_4], **(c)** [MBA][HSO_4] and **(d)** [DMBA][HSO_4], obtained from the classical molecular dynamics simulations of the respective anhydrous (blue) and doped with 20 wt % water (green) protic ILs. Size distribution data was computed using the AGGREGATES software, employing the corresponding S-S distance cut-off determined from the first minimum of the anion-anion radial distribution functions.

E. Supplementary *Ab Initio* Molecular Dynamics Simulation Results

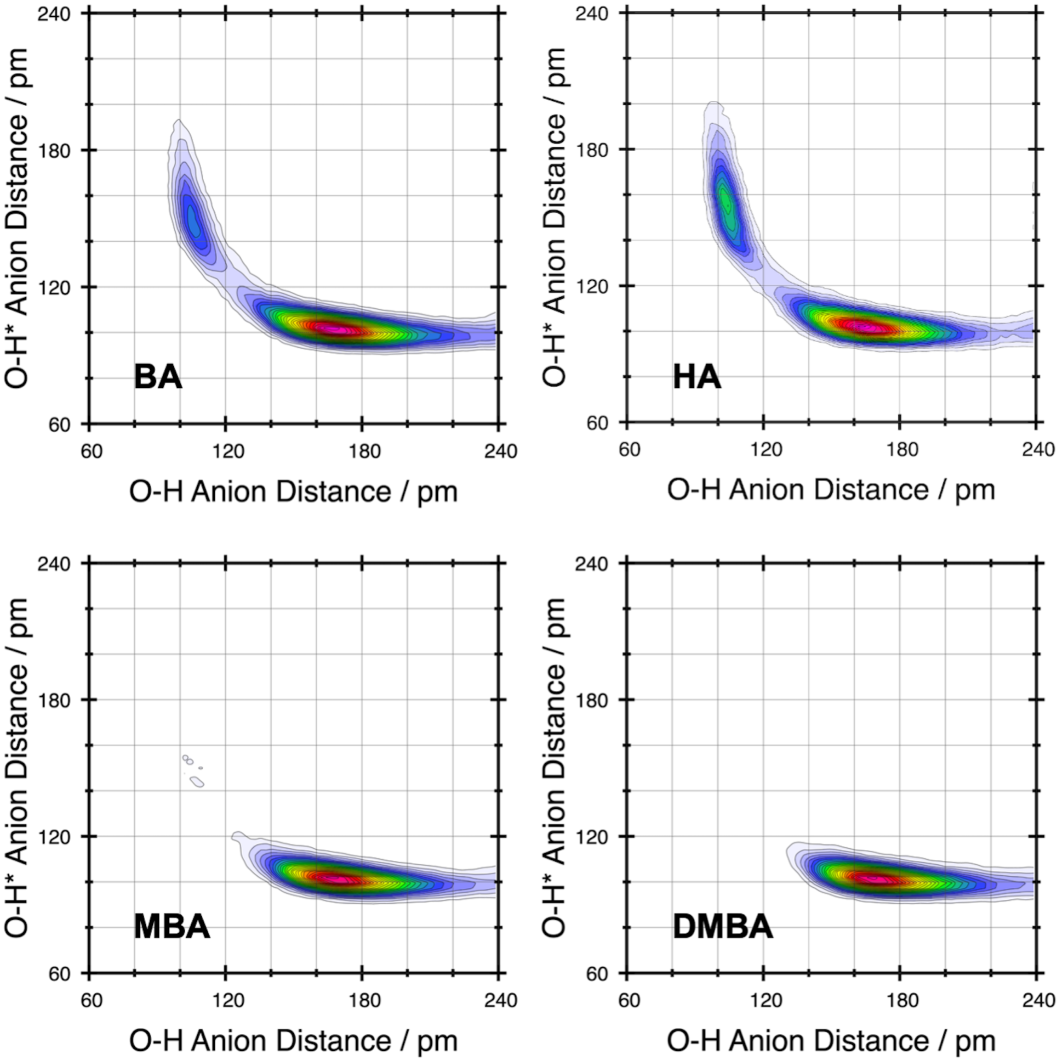

Figure S10: Combined RDFs of anion O···H vs. anion O···H* distance. Where H* originates from the acidic $[HSO_4]^-$ anion.

F. Supplementary Quantum Chemical (DFT) Results

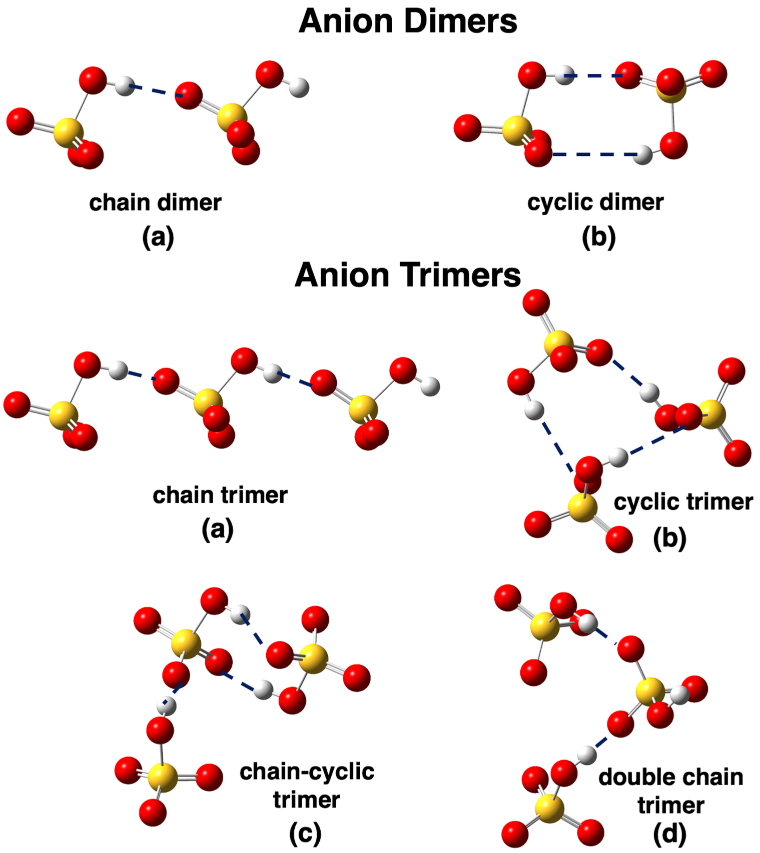

Figure S11: Anion dimer and trimer configurations and corresponding anion-anion interactions observed from classical molecular dynamics simulations of anhydrous and aqueous [BA][HSO_4], [HA][HSO_4], [MBA][HSO_4] and [DMBA][HSO_4].

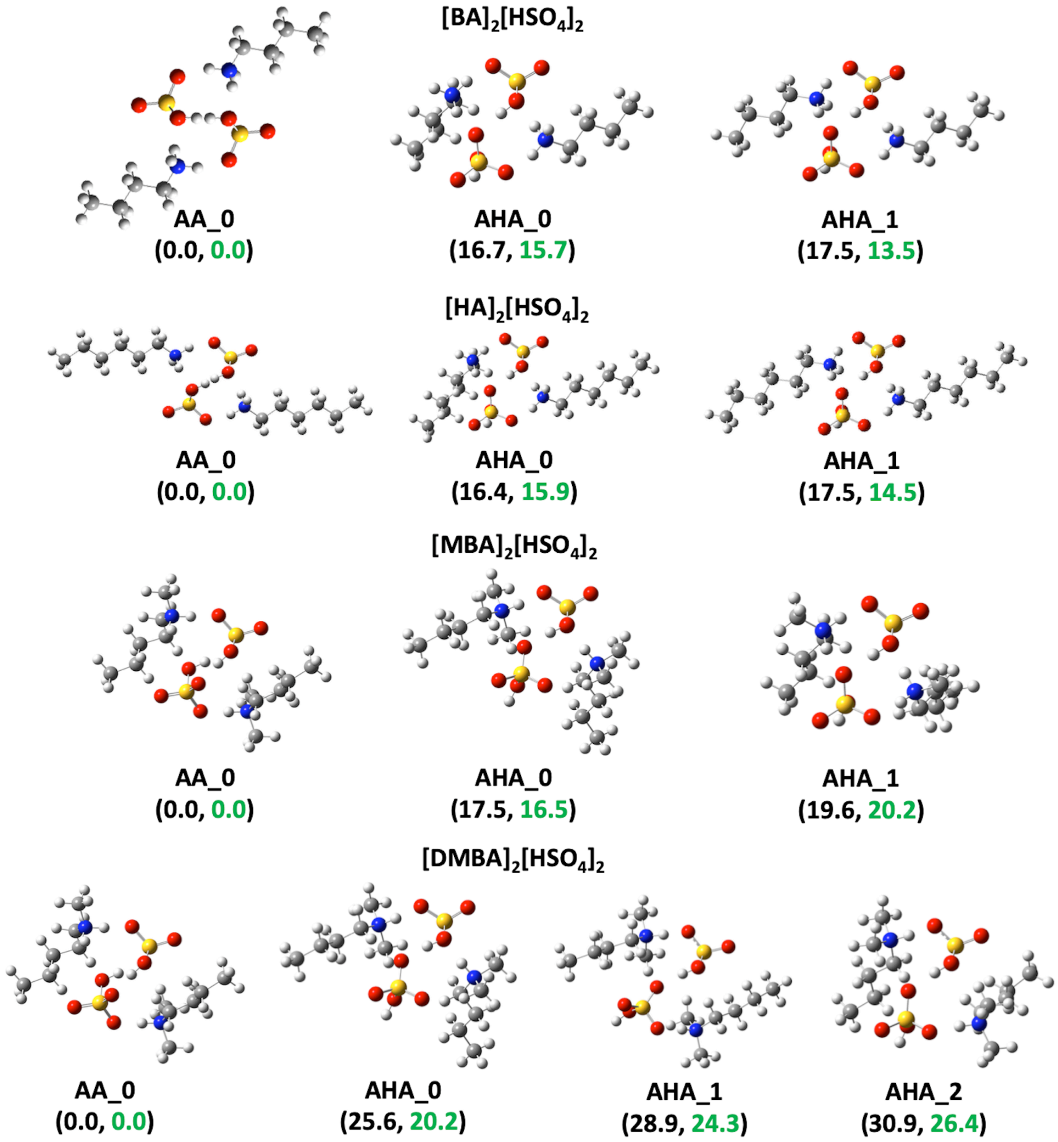

Figure S12: Representative lowest energy structures for the anhydrous Protic IL ion pair dimers. In these structures the two $[HSO_4]^-$ anions are found to orientate in either a cyclic (**AA** – contains two anion-anion hydrogen bonds) or chain (**AHA** – 1 x anion-anion hydrogen bond) conformation (see **Figure S11**). ΔE_{ZPE} (black) and ΔG (green) energies are all reported in kJ.mol^{-1}.

Table S3: Energetic data for the lowest energy structures for the anhydrous PIL ion pair dimers in the gas-phase. These structures are labelled according to the orientation of the two $[HSO_4]^-$ anions – either cyclic (**AA** – contains two anion-anion hydrogen bonds) or chain (**AHA** – 1 x anion-anion hydrogen bond). See **Figure S12** for a clearer view of these structural conformers. All energy differences are reported in kJ.mol^{-1} and ΔE_{ZPE} includes the ZPE correction. All of E, G, H, S and ZPE have been rounded to fifth decimal point.

IL	Structural Conformer	ΔE_{ZPE} (kJ.mol^{-1})	ΔG (kJ.mol^{-1})	E (a.u.)	G (a.u.)	H (a.u.)	S (a.u.)	ZPE (a.u.)
$[BA]_2[HSO_4]_2$	**AA_0**	0.00	0.00	-1828.62111	-1828.2959	-1828.20558	0.09032	0.38809
	**AHA_0**	16.69	15.72	-1828.61366	-1828.28992	-1828.19854	0.09137	0.38699
	**AHA_1**	17.54	15.84	-1828.61329	-1828.29075	-1828.19799	0.09276	0.38695
$[HA]_2[HSO_4]_2$	**AA_0**	0.00	0.00	-1985.93792	-1985.50863	-1985.4035	0.10513	0.50149
	**AHA_0**	16.45	15.88	-1985.93056	-1985.50258	-1985.39656	0.10602	0.50040
	**AHA_1**	17.49	14.49	-1985.93016	-1985.50311	-1985.39594	0.10717	0.50039
$[MBA]_2[HSO_4]_2$	**AA_0**	0.00	0.00	-1907.27436	-1906.89565	-1906.79926	0.09639	0.44461
	**AHA_0**	17.51	16.47	-1907.26663	-1906.88938	-1906.79181	0.09757	0.44355
	**AHA_1**	19.55	20.18	-1907.26593	-1906.88797	-1906.79119	0.09678	0.44364
$[DMBA]_2[HSO_4]_2$	**AA_0**	0.00	0.00	-1985.90708	-1985.4742	-1985.37368	0.10052	0.50029
	**AHA_0**	25.60	20.22	-1985.89672	-1985.4665	-1985.36304	0.10346	0.49968
	**AHA_1**	28.86	24.30	-1985.89527	-1985.46495	-1985.36183	0.10312	0.49947
	**AHA_2**	30.91	26.41	-1985.89436	-1985.46414	-1985.36107	0.10307	0.49933

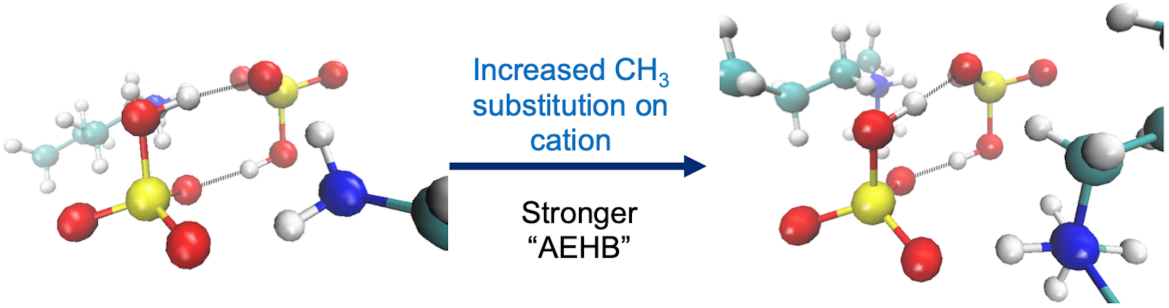

	r_{OH-O} (Å)	ρ_b (au)	$\nabla^2\rho_b$ (au)	H_b (au)	E(2) (kcal mol^{-1})
**$[BA]_2[HSO_4]_2$**	1.685	0.046	0.142	-0.0043	26.8
**$[HA]_2[HSO_4]_2$**	1.685	0.046	0.142	-0.0042	26.8
**$[MBA]_2[HSO_4]_2$**	1.671	0.047	0.145	-0.0050	28.2
**$[DMBA]_2[HSO_4]_2$**	1.611	0.055	0.158	-0.0086	36.9
	(1.645)	(0.048)	(0.152)	-0.0053	(30.3)

Figure S13: QTAIM and NBO data for the Anion-Anion H-Bonding interactions found for the lowest energy structure (**AA_0** – **Table S3**) for each of the anhydrous PIL ion pair dimers.

Table S4: Energetic data for the lowest energy structures for the protic IL ion pair dimers containing 6 H_2O molecules (~20 wt% H_2O) and include the solvent environment via CPCM (ethanol). These structures are identified according to the orientation of the two $[HSO_4]^-$ anions and waters - cyclic (**AA** – contains two anion-anion hydrogen bonds), chain (**AHA** – 1 x anion-anion hydrogen bond) intercalated waters (**IC** – 2 x waters between the anions) and proton transfer (**PT**). All energy differences are reported in $kJ.mol^{-1}$ and ΔE_{ZPE} includes the ZPE correction. All of E, G, H, S and ZPE have been rounded to fifth decimal point.

IL	Structural Conformer	ΔE ($kJ.mol^{-1}$)	ΔG ($kJ.mol^{-1}$)	E (a.u.)	G (a.u.)	H (a.u.)	TΔS (a.u.)	ZPE (a.u.)
	**AA_0**	0.00	0.00	-2287.557533	-2287.101692	-2286.975944	0.125748	0.537539
	**AHA_0**	3.59	4.40	-2287.555394	-2287.100015	-2286.974882	0.125133	0.536768
	**AA_1**	12.18	5.68	-2287.551567	-2287.099529	-2286.970396	0.129133	0.536211
**$[BA]_2[HSO_4]_2$**	**AHA_1**	11.80	9.59	-2287.552585	-2287.098039	-2286.971417	0.126622	0.537086
	**IC_0**	30.33	32.68	-2287.547137	-2287.089244	-2286.965037	0.124207	0.538696
	**PT_0**	1.55	4.62	-2287.557296	-2287.099933	-2286.977029	0.122904	0.537891
	**PT_1**	20.01	28.15	-2287.550321	-2287.090969	-2286.96987	0.121099	0.5379507
	**AA_0**	0.00	0.00	-2444.87445	-2444.314101	-2444.174164	0.139937	0.65077
	**AHA_0**	2.94	2.33	-2444.872397	-2444.313214	-2444.173277	0.139937	0.649835
	**AA_1**	10.87	1.62	-2444.869243	-2444.313484	-2444.1691	0.144384	0.649703
**$[HA]_2[HSO_4]_2$**	**AHA_1**	11.84	10.24	-2444.869507	-2444.310201	-2444.169629	0.140572	0.650335
	**AA_2**	14.43	8.86	-2444.867858	-2444.310728	-2444.167764	0.142964	0.649673
	**IC_0**	28.95	33.74	-2444.864855	-2444.301249	-2444.163816	0.137433	0.652203
	**PT_0**	0.96	3.51	-2444.874313	-2444.312763	-2444.175397	0.137366	0.650997
	**PT_1**	18.36	23.70	-2444.867291	-2444.305075	-2444.168379	0.136696	0.650603
	**AA_0**	0.00	0.00	-2366.198225	-2365.691564	-2365.556953	0.134611	0.593655
	**AHA_0**	3.46	9.38	-2366.19722	-2365.687991	-2365.556009	0.131982	0.593968
**$[MBA]_2[HSO_4]_2$**	**AA_1**	9.08	-4.67	-2366.192469	-2365.693343	-2365.552098	0.141245	0.591356
	**IC_0**	11.34	13.49	-2366.19369	-2365.686425	-2365.553162	0.133263	0.593441
	**PT_0**	-1.94	11.95	-2366.200089	-2365.687013	-2365.559917	0.127096	0.594779
	**PT_1**	-0.70	10.15	-2366.199064	-2365.687698	-2365.558908	0.12879	0.594226
	**AHA_0**	0.00	0.00	-2444.830581	-2444.271024	-2444.131359	0.139665	0.648633
	**AA_0**	1.66	3.56	-2444.831216	-2444.269669	-2444.131393	0.138276	0.6499
	**AA_1**	13.76	6.15	-2444.825274	-2444.268682	-2444.125494	0.143188	0.648566
**$[DMBA]_2[HSO_4]_2$**	**IC_0**	18.80	20.12	-2444.825799	-2444.263361	-2444.125294	0.138067	0.651012
	**PT_0**	-5.42	-1.37	-2444.833906	-2444.271546	-2444.13495	0.136596	0.649894
	**PT_1**	-0.18	6.33	-2444.831905	-2444.268613	-2444.133082	0.135531	0.649889

Table S5: Energetic data for the two low energy structures where proton transfer (X_PT0; X is the parent cation, i.e. BA, HA, MBA and DMBA) has occurred or where proton transfer has not occurred (X_0) for the protic IL ion pair trimers containing 9 H_2O molecules (~20 wt% H_2O) and include the solvent environment via CPCM (ethanol). All energy differences are reported in kJ.mol^{-1} and ΔE_{ZPE} includes the ZPE correction. All of E, G, H, S and ZPE have been rounded to fifth decimal point.

IL	Structural Conformer	ΔE (kJ.mol^{-1})	ΔG (kJ.mol^{-1})	E (a.u.)	G (a.u.)	H (a.u.)	S (a.u.)	ZPE (a.u.)
$[BA]_3[HSO_4]_3$	BA_PT0	-3.75	-7.24	-3431.35966	-3430.65988	-3430.48714	0.17273	0.80779
	BA_0	0.00	0.00	-3431.35966	-3430.65712	-3430.48512	0.17200	0.80922
$[HA]_3[HSO_4]_3$	HA_PT0	-6.93	-9.89	-3667.33683	-3666.47934	-3666.28622	0.19312	0.97774
	HA_0	0.00	0.00	-3667.33578	-3666.47557	-3666.28305	0.19253	0.97933
$[MBA]_3[HSO_4]_3$	MBA_PT0	0.37	13.46	-3549.32329	-3548.54011	-3548.36072	0.17939	0.89353
	MBA_0	0.00	0.00	-3549.32303	-3548.54523	-3548.35973	0.18550	0.89313
$[DMBA]_3[HSO_4]_3$	DMBA_PT0	-18.37	-8.15	-3667.27166	-3666.41330	-3666.22181	0.19149	0.97636
	DMBA_0	0.00	0.00	-3667.26402	-3666.41019	-3666.21243	0.19777	0.97572

References:

(1) Gräsvik, J.; Hallett, J. P.; To, T. Q.; Welton, T. A quick, simple, robust method to measure the acidity of ionic liquids. *Chem. Commun.* **2014**, *50* (55), 7258-7261. DOI: 10.1039/c4cc02816c.

(2) Hammett, L. P.; Deyrup, A. J. A series of simple basic indicators. I. The acidity functions of mixtures of dulfuric and perchloric acids with water1. *J. Am. Chem. Soc.* **2002**, *54* (7), 2721-2739. DOI: 10.1021/ja01346a015.

(3) Stewart, R.; Dolman, D. A comparison of the acidity and basicity of aromatic amines. *Can. J. Chem.* **1967**, *45* (9), 925-928. DOI: 10.1139/v67-156.

(4) D.A. Case, R. M. B., D.S. Cerutti, T.E. Cheatham, III, T.A. Darden, R.E. Duke, T.J. Giese, H. Gohlke, A.W. Goetz, N. Homeyer, S. Izadi, P. Janowski, J. Kaus, A. Kovalenko, T.S. Lee, S. LeGrand, P. Li, C. Lin, T. Luchko, R. Luo, B. Madej, D. Mermelstein, K.M. Merz, G. Monard, H. Nguyen, H.T. Nguyen, I. Omelyan, A. Onufriev, D.R. Roe, A. Roitberg, C. Sagui, C.L. Simmerling, W.M. Botello-Smith, J. Swails, R.C. Walker, J. Wang, R.M. Wolf, X. Wu, L. Xiao and P.A. Kollman. AMBER 2016. University of California, San Francisco: 2016.

(5) Brehm, M.; Kirchner, B. TRAVIS - a free analyzer and visualizer for Monte Carlo and molecular dynamics trajectories. *J Chem Inf Model* **2011**, *51* (8), 2007-2023. DOI: 10.1021/ci200217w.

(6) Brehm, M.; Thomas, M.; Gehrke, S.; Kirchner, B. TRAVIS-A free analyzer for trajectories from molecular simulation. *J. Chem. Phys.* **2020**, *152* (16), 164105. DOI: 10.1063/5.0005078.

(7) Bernardes, C. E. AGGREGATES: Finding structures in simulation results of solutions. *J. Comput. Chem.* **2017**, *38* (10), 753-765. DOI: 10.1002/jcc.24735.

(8) Roe, D. R.; Cheatham, T. E., 3rd. PTRAJ and CPPTRAJ: Software for processing and analysis of molecular dynamics trajectory data. *J. Chem. Theory Comput.* **2013**, *9* (7), 3084-3095. DOI: 10.1021/ct400341p.

(9) Wang, J.; Wolf, R. M.; Caldwell, J. W.; Kollman, P. A.; Case, D. A. Development and testing of a general amber force field. *J. Comput. Chem.* **2004**, *25* (9), 1157-1174. DOI: 10.1002/jcc.20035.

(10) Horn, H. W.; Swope, W. C.; Pitera, J. W.; Madura, J. D.; Dick, T. J.; Hura, G. L.; Head-Gordon, T. Development of an improved four-site water model for biomolecular simulations: TIP4P-Ew. *J. Chem. Phys.* **2004**, *120* (20), 9665-9678. DOI: 10.1063/1.1683075.

(11) Fedorova, I. V.; Krestyaninov, M. A.; Safonova, L. P. Structure of ethylammonium hydrogen sulfate protic ionic liquid through DFT calculations and MD simulations: the role of hydrogen bonds. *Struct. Chem.* **2022**, *34* (3), 879-890. DOI: 10.1007/s11224-022-02042-7.

(12) Sprenger, K. G.; Jaeger, V. W.; Pfaendtner, J. The general AMBER force field (GAFF) can accurately predict thermodynamic and transport properties of many ionic liquids. *J. Phys. Chem. B* **2015**, *119* (18), 5882-5895. DOI: 10.1021/acs.jpcb.5b00689.

(13) Tenney, C. M.; Massel, M.; Mayes, J. M.; Sen, M.; Brennecke, J. F.; Maginn, E. J. A computational and experimental study of the heat transfer properties of nine different ionic liquids. *J. Chem. Eng. Data* **2014**, *59* (2), 391-399. DOI: 10.1021/je400858t.

(14) Woods, R. J.; Chappelle, R. Restrained electrostatic potential atomic partial charges for condensed-phase simulations of carbohydrates. *THEOCHEM* **2000**, *527* (1-3), 149-156. DOI: 10.1016/S0166-1280(00)00487-5.

(15) Barbosa, N. S. V.; Zhang, Y.; Lima, E. R. A.; Tavares, F. W.; Maginn, E. J. Development of an AMBER-compatible transferable force field for poly(ethylene glycol) ethers (glymes). *J. Mol. Model.* **2017**, *23* (6), 194. DOI: 10.1007/s00894-017-3355-3.

(16) Martinez, L.; Andrade, R.; Birgin, E. G.; Martinez, J. M. PACKMOL: a package for building initial configurations for molecular dynamics simulations. *J. Comput. Chem.* **2009**, *30* (13), 2157-2164. DOI: 10.1002/jcc.21224.

(17) Darden, T.; York, D.; Pedersen, L. Particle mesh Ewald: An N·log(N) method for Ewald sums in large systems. *J. Chem. Phys.* **1993**, *98* (12), 10089-10092. DOI: 10.1063/1.464397.

(18) Pastor, R. W.; Brooks, B. R.; Szabo, A. An analysis of the accuracy of Langevin and molecular dynamics algorithms. *Mol. Phys.* **2006**, *65* (6), 1409-1419. DOI: 10.1080/00268978800101881.

(19) Berendsen, H. J. C.; Postma, J. P. M.; van Gunsteren, W. F.; DiNola, A.; Haak, J. R. Molecular dynamics with coupling to an external bath. *J. Chem. Phys.* **1984**, *81* (8), 3684-3690. DOI: 10.1063/1.448118.
(20) Ryckaert, J.-P.; Ciccotti, G.; Berendsen, H. J. C. Numerical integration of the cartesian equations of motion of a system with constraints: molecular dynamics of n-alkanes. *J. Comput. Phys.* **1977**, *23* (3), 327-341. DOI: 10.1016/0021-9991(77)90098-5.
(21) Matthews, R. P.; Villar-Garcia, I. J.; Weber, C. C.; Griffith, J.; Cameron, F.; Hallett, J. P.; Hunt, P. A.; Welton, T. A structural investigation of ionic liquid mixtures. *Phys. Chem. Chem. Phys.* **2016**, *18* (12), 8608-8624. DOI: 10.1039/c6cp00156d.
(22) VandeVondele, J.; Krack, M.; Mohamed, F.; Parrinello, M.; Chassaing, T.; Hutter, J. Quickstep: Fast and accurate density functional calculations using a mixed Gaussian and plane waves approach. *Comput. Phys. Commun.* **2005**, *167* (2), 103-128. DOI: 10.1016/j.cpc.2004.12.014.
(23) Kuhne, T. D.; Iannuzzi, M.; Del Ben, M.; Rybkin, V. V.; Seewald, P.; Stein, F.; Laino, T.; Khaliullin, R. Z.; Schutt, O.; Schiffmann, F.; et al. CP2K: An electronic structure and molecular dynamics software package - Quickstep: Efficient and accurate electronic structure calculations. *J. Chem. Phys.* **2020**, *152* (19), 194103. DOI: 10.1063/5.0007045.
(24) Becke, A. D. Density-functional exchange-energy approximation with correct asymptotic behavior. *Phys Rev A Gen Phys* **1988**, *38* (6), 3098-3100. DOI: 10.1103/physreva.38.3098.
(25) Perdew, J. P. Density-functional approximation for the correlation energy of the inhomogeneous electron gas. *Phys Rev B Condens Matter* **1986**, *33* (12), 8822-8824. DOI: 10.1103/physrevb.33.8822.
(26) VandeVondele, J.; Hutter, J. Gaussian basis sets for accurate calculations on molecular systems in gas and condensed phases. *J. Chem. Phys.* **2007**, *127* (11), 114105. DOI: 10.1063/1.2770708.
(27) Goedecker, S.; Teter, M.; Hutter, J. Separable dual-space Gaussian pseudopotentials. *Phys Rev B Condens. Matter* **1996**, *54* (3), 1703-1710. DOI: 10.1103/physrevb.54.1703.
(28) Hartwigsen, C.; Goedecker, S.; Hutter, J. Relativistic separable dual-space Gaussian pseudopotentials from H to Rn. *Phys Rev B Condens. Matter* **1998**, *58* (7), 3641-3662. DOI: 10.1103/PhysRevB.58.3641.
(29) Krack, M. Pseudopotentials for H to Kr optimized for gradient-corrected exchange-correlation functionals. *Theor. Chem. Acc.* **2005**, *114* (1-3), 145-152. DOI: 10.1007/s00214-005-0655-y.
(30) Grimme, S.; Antony, J.; Ehrlich, S.; Krieg, H. A consistent and accurate ab initio parametrization of density functional dispersion correction (DFT-D) for the 94 elements H-Pu. *J. Chem. Phys.* **2010**, *132* (15), 154104. DOI: 10.1063/1.3382344.
(31) Grimme, S.; Ehrlich, S.; Goerigk, L. Effect of the damping function in dispersion corrected density functional theory. *J. Comput. Chem.* **2011**, *32* (7), 1456-1465. DOI: 10.1002/jcc.21759.
(32) Thomas, M.; Brehm, M.; Holloczki, O.; Kirchner, B. How can a carbene be active in an ionic liquid? *Chemistry* **2014**, *20* (6), 1622-1629. DOI: 10.1002/chem.201303329.
(33) Nosé, S. A unified formulation of the constant temperature molecular dynamics methods. *J. Chem. Phys.* **1984**, *81* (1), 511-519. DOI: 10.1063/1.447334.
(34) Hoover, W. G. Canonical dynamics: Equilibrium phase-space distributions. *Phys Rev A Gen Phys* **1985**, *31* (3), 1695-1697. DOI: 10.1103/physreva.31.1695.
(35) Frisch, M. J.; Trucks, G. W.; Schlegel, H. B.; Scuseria, G. E.; Robb, M. A.; Cheeseman, J. R.; Scalmani, G.; Barone, V.; Petersson, G. A.; Nakatsuji, H.; et al. Gaussian 16 Rev. C.01. Wallingford, CT, 2016.
(36) Lee, C.; Yang, W.; Parr, R. G. Development of the Colle-Salvetti correlation-energy formula into a functional of the electron density. *Phys Rev B Condens. Matter* **1988**, *37* (2), 785-789. DOI: 10.1103/physrevb.37.785.
(37) Becke, A. D.; Johnson, E. R. Exchange-hole dipole moment and the dispersion interaction. *J. Chem. Phys.* **2005**, *122* (15), 154104. DOI: 10.1063/1.1884601.
(38) Becke, A. D.; Johnson, E. R. Exchange-hole dipole moment and the dispersion interaction: high-order dispersion coefficients. *J. Chem. Phys.* **2006**, *124* (1), 14104. DOI: 10.1063/1.2139668.
(39) Johnson, E. R.; Becke, A. D. A post-Hartree-Fock model of intermolecular interactions. *J. Chem. Phys.* **2005**, *123* (2), 24101. DOI: 10.1063/1.1949201.

(40) Barone, V.; Cossi, M. Quantum calculation of molecular energies and energy gradients in solution by a conductor solvent model. *J. Phys. Chem. A* **1998**, *102* (11), 1995-2001. DOI: 10.1021/jp9716997.
(41) Cossi, M.; Rega, N.; Scalmani, G.; Barone, V. Energies, structures, and electronic properties of molecules in solution with the C-PCM solvation model. *J. Comput. Chem.* **2003**, *24* (6), 669-681. DOI: 10.1002/jcc.10189.
(42) Low, K.; Tan, S. Y. S.; Izgorodina, E. I. An ab initio study of the structure and energetics of hydrogen bonding in ionic liquids. *Front Chem* **2019**, *7*, 208. DOI: 10.3389/fchem.2019.00208.
(43) Bader, R. F. W. A quantum theory of molecular structure and its applications. *Chem. Rev.* **2002**, *91* (5), 893-928. DOI: 10.1021/cr00005a013.
(44) Keith, T. A. AIMALL (Version 19.10.12). TK Gristmill Software, Overland Park, KS, USA, 2019.
(45) Reed, A. E.; Curtiss, L. A.; Weinhold, F. Intermolecular interactions from a natural bond orbital, donor-acceptor viewpoint. *Chem. Rev.* **2002**, *88* (6), 899-926. DOI: 10.1021/cr00088a005.
(46) Glendening, E. D.; Landis, C. R.; Weinhold, F. NBO 6.0: natural bond orbital analysis program. *J. Comput. Chem.* **2013**, *34* (16), 1429-1437. DOI: 10.1002/jcc.23266.
(47) Sedov, I. A.; Magsumov, T. I. Solvation properties of protic ionic liquids 2-methoxyethylammonium nitrate, propylammonium hydrogen sulfate, and butylammonium hydrogen sulfate. *Journal Chem. Thermodyn.* **2022**, *170*. DOI: 10.1016/j.jct.2022.106779.
